# Biotransformation of Corn-Derived *N* ^1^,*N* ^10^-di‑*p*‑Coumaroyl Spermidine in Mice and by Human Gut Microbiota Reveals Novel Reduced Metabolites

**DOI:** 10.1021/acs.jafc.6c06580

**Published:** 2026-08-03

**Authors:** Salar Hafez-Ghoran, Nathaniel Yeboah, Shengmin Sang

**Affiliations:** † Laboratory for Functional Foods and Human Health, Center for Excellence in Post-Harvest Technologies, 3616 North Carolina Agricultural and Technical State University, North Carolina Research Campus, 500 Laureate Way, Kannapolis, North Carolina 28081, United States; ‡ Department of Chemistry, 3616 North Carolina Agricultural and Technical State University, Greensboro, North Carolina 27411, United States

**Keywords:** *Zea mays* L., corn phenolamides, *N* ^1^,*N* ^10^-di-p-coumaroyl spermidine, in vivo biotransformation, human gut microbiota

## Abstract

While candidate biomarkers have been proposed for several cereals and pseudocereals, no validated biomarkers have been established for whole grain (WG) corn. As an initial step toward biomarker development, this study investigated the biotransformation of *N* ^1^,*N* ^10^-di-*p*-coumaroyl spermidine (diCouSpd), a major corn phenolamide, using an integrated approach combining chemical synthesis, LC-MS- and NMR-based structural elucidation, *in vitro* human fecal fermentation, and *in vivo* mouse studies. Three previously unreported hydrogenated metabolites were identified: *N* ^1^-dihydro-*p*-coumaroyl-*N* ^10^-*p*-coumaroyl-spermidine (**1**), *N* ^1^-*p*-coumaroyl-*N* ^10^-dihydro-*p*-coumaroyl-spermidine (**2**), and *N* ^1^,*N* ^10^-bis­(dihydro-*p*-coumaroyl)-spermidine (**3**). Fecal and urine analyses from mice administrated diCouSpd or corn extracts prepared from two or four servings of WG corn confirmed the formation of these reduced metabolites. Human fecal fermentation revealed a putative stepwise hydrogenation pathway involving sequential reduction of *p*-coumaroyl moieties in diCouSpd. Collectively, these findings provide new insights into the reductive metabolism of corn phenolamides and support diCouSpd and its metabolites as potential exposure biomarkers of WG corn intake.

## Graphical abstract

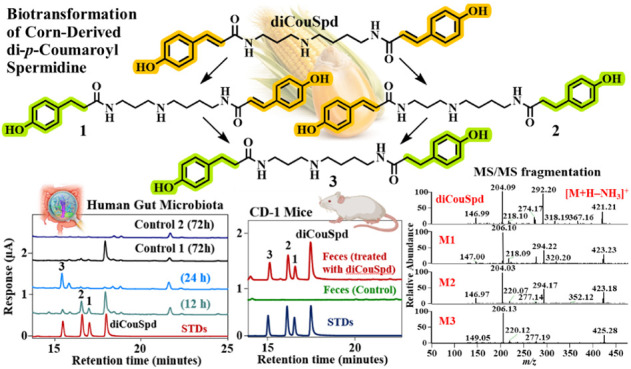

## 1 Introduction

The 2025–2030 Dietary Guidelines for Americans recognize whole grains (WGs) as key components of a healthy diet due to their contribution of essential macro- and micronutrients.^1^ Corn (*Zea mays* L., Poaceae) is a globally important cereal crop and a major component of WG diets consumed by over one billion people worldwide. Epidemiological evidence associates regular intake of WG corn with reduced risk of chronic diseases, including cardiovascular disease, type 2 diabetes, and obesity, as well as improved gastrointestinal health.^2^ However, elucidating the mechanisms underlying these health effects requires more precise dietary assessment tools, particularly biomarkers that reflect intake of specific cereal foods. While candidate biomarkers have been proposed for several cereals and pseudocereals and for different grain fractions,^3^ no validated biomarkers have been established for corn. This gap is further complicated by the extensive metabolism of dietary compounds by host and gut microbiota, and the lack of human intervention studies, which together limit current understanding of the metabolic fate of corn-derived phytochemicals.

Beyond macronutrients, WG corn contains a diverse array of phytochemicals, including carotenoids, tocopherols, sterols, phenolics, flavonoids, and phenolamides, whose composition varies with genotype, grain fraction, and pigmentation.^4^ Among these, phenolamides (hydroxycinnamic acid amides) are conjugates of hydroxycinnamic acids with biogenic amines, occurring in both free and bound forms.^5^ These compounds have attracted increasing attention due to their antioxidant^6^ and anti-inflammatory activities, as well as their potential to modulate gut microbiota.^7^ Conjugated phenolamides often exhibit higher bioactivity than their corresponding free hydroxycinnamic acids and polyamines, including reported inhibition of HIV-1 protease.^8^

Based on the current literature, *N* ^1^,*N* ^10^-di-*p*-coumaroyl spermidine (diCouSpd) has been exclusively reported in corn and has not been identified in other foods.^4,7^ Despite its relatively high abundance in corn, the metabolic fate of diCouSpd following dietary intake remains unknown. Therefore, the present study aimed to (i) identify the major metabolites of diCouSpd *in vivo* using a mouse model, (ii) elucidate their chemical structures through targeted chemical synthesis combined with HR-LC-MS and 1D/2D NMR analyses, (iii) evaluate human dietary-relevant exposure through oral administration of corn extract at levels corresponding to typical dietary intake, and (iv) investigate the *in vitro* biotransformation of diCouSpd using human fecal slurry. This work provides new insights into the biotransformation of corn phenolamides and serves as an initial step toward evaluating the potential of diCouSpd and its metabolites as candidate biomarkers of WG corn intake.

## 2 Materials and Methods

### 2.1 Chemicals and Reagents

Chloroform (CHCl_3_), methanol (MeOH), acetonitrile (ACN), tetrahydrofuran (THF), deionized water (DW), and formic acid (FA) (ACS-, HPLC-, or LC-MS-grade, as appropriate) were obtained from Thermo Fisher Scientific (Petersburg, USA). Silica gel G60 (350–600 mesh), Sephadex LH-20, 4-dimethylaminopyridine (DMAP), *N*,*N′*-dicyclohexylcarbodiimide (DCC), dimethylformamide (DMF), deuterated methanol (CD_3_OD), *p*-coumaric acid (*p*-CA), dihydro-*p*-coumaric acid (diH-*p*-CA), spermidine hydrochloride, phosphoric acid, monosodium phosphate, sodium acetate anhydrous (NaOAc), ascorbic acid, and β-glucuronidase/sulfatase were purchased from Sigma-Aldrich (St. Louis, MO, USA). Gifu anaerobic broth (GAM) medium was obtained from HIMEDIA Laboratories (Mumbai, India). *N* ^1^,*N* ^10^-Di-*p*-coumaroyl spermidine (diCouSpd) was synthesized in-house following literature method.^9^

### 2.2 HPLC-ECD and LC-MS Analysis

HPLC with electrochemical detection (HPLC-ECD) was performed using an ESA system equipped with dual Model 584 pumps, a Model 542 autosampler, and a Model 5600A CoulArray detector coupled with two Model 6210 four-sensor cells. Mobile phase A consisted of deionized water (1962.5 mL), ACN (35 mL), and THF (2.5 mL), containing phosphoric acid (250 μL) and monosodium phosphate (8.29 g). Mobile phase B comprised deionized water (580 mL), ACN (1170 mL), THF (250 mL), with phosphoric acid (1.25 mL) and monosodium phosphate (4.17 g). Chromatographic separation was achieved on a Gemini C18 column (150 × 4.6 mm i.d., 5 μm; Phenomenex) at a flow rate of 1.5 mL/min with an injection volume of 10 μL. The gradient program was as follows: 0–15% B (0–10 min), 15–35% B (10–20 min), 35–50% B (20–25 min), 50–100% B (25–30 min), 100% B (30–34 min), followed by re-equilibration at 0% B (34–38 min). Electrochemical detection was conducted using a colorimetric electrode array with applied potentials of 50, 100, 200, 300, 400, 500, 600, and 700 mV. Data were acquired from the 400 mV channel.

Targeted LC-MS/MS analyses for metabolite discovery were performed using a UHPLC system (Dionex Ultimate 3000; Thermo Fisher Scientific coupled to a linear ion trap mass spectrometer (LTQ Velos Pro) equipped with an ESI source operating in positive ion mode. Chromatographic conditions were identical to those described above, except for an injection volume of 5 μL. Mass spectrometric detection was carried out using selected ion monitoring (SIM) over an *m*/*z* range of 50–1000. MS/MS spectra were acquired using collision-induced dissociation (CID; normalized collision energy = 35 eV) for structural confirmation. Source parameters were: sheath gas (N_2_), 29 arbitrary units; auxiliary gas, 16 arbitrary units; sweep gas, 0 arbitrary units; capillary temperature 300 °C; spray voltage 3.60 kV; and spray current, 100 μA. Data acquisition and processing were performed using Xcalibur software (Thermo Fisher Scientific).

High-resolution LC-MS/MS analyses for metabolite accurate mass measurements were performed using a Thermo Fisher Scientific system consisting of a Dionex Ultimate 3000 RS pump and autosampler coupled to a Q Exactive Plus Orbitrap mass spectrometer equipped with an electrospray ionization (ESI) source (Waltham, USA). Chromatographic separation was performed on a reverse-phase Gemini C18 column (50 × 2.0 mm × 3 μm, 110 Å; Phenomenex) maintained at 40 °C. The mobile phases consisted of 0.1% FA in water (A) and 0.1% FA in ACN (B). The gradient program was: 2% B (0–1 min), 2–25% B (1–3 min), 25–30% B (3–4 min), 30–40% B (4–7.5 min), 40–50% B (7.5–8.0 min), 50–100% B (8–8.5 min), 100% B (8.5–13.5 min), followed by re-equilibration at 2% B (13.5–16 min). The flow rate was 0.25 mL/min and the injection volume was 3 μL. The mass spectrometer was operated in both positive and negative ion modes using fullMS/ddMS^2^ over an *m*/*z* range of 100–1500. For positive ion mode, parameters were: sheath gas (N_2_), 30 arbitrary units; auxiliary gas, 12 arbitrary units; sweep gas, 3 arbitrary units; capillary temperature 295 °C; spray voltage 3.65 kV; spray current, 100 μA; and S-lens RF level, 60. For negative ion mode, parameters were: sheath gas (N_2_), 32 arbitrary units; auxiliary gas 18 arbitrary units; sweep gas 0 arbitrary units; capillary temperature 250 °C; spray voltage 2.6 kV; spray current, 100 μA; and S-lens RF level, 60. Full MS scans were acquired at a resolution of 70,000 fwhm, and MS/MS scans at 17,500 fwhm using stepped normalized collision energies of 10, 40, 70 eV. Data acquisition and processing were performed using Xcalibur software (Thermo Fisher Scientific).

### 2.3 One-Pot Synthesis, Purification, and Characterization of diCouSpd Metabolites

The metabolites of diCouSpd, **1**-**3**, were synthesized via a one-pot DCC/DMAP-mediated coupling strategy,^9^ with minor modifications. In brief, DCC (1.1 mmol; 226.9 mg) and a mixture of *p*-CA (0.5 mmol, 82.0 mg) and diH-*p*-CA (0.5 mmol, 83.8 mg) were dissolved in DMF and stirred at room temperature for 1 h. In a separate flask, spermidine trihydrochloride (0.5 mmol; 126.6 mg) and DMAP (0.2 mM; 24.2 mg) were dissolved in DMF and added dropwise to the activated acid solution. The reaction was stirred at room temperature for 24 h. The reaction was quenched with deionized water (15 mL), and the solvents were removed under reduced pressure using a rotary evaporator. The resulting residue was triturated with MeOH (3×) and the combined MeOH portions were mixed with silica gel G60 (3 g) and evaporated to dryness to afford a free-flowing powder suitable for vacuum liquid chromatography (VLC).

The silica-absorbed crude product was fractionated by VLC (150 mL sintered funnel; silica gel G60 350–600 mesh, 30 g) using a CHCl_3_:MeOH gradient (10:0, 9:1, 8:2, 7:3, 6:4, 5:5, 4:6, 2.5:7.5, and 0:10; 200 mL each), yielding nine fractions A–I. Fractions E and F, contained diCouSpd and its metabolites **1**–**3**, were compiled and further purified by semipreparative HPLC-DAD using an Agilent 1260 Infinity II system equipped with a binary pump (G7112B), autosampler (G7129A), and diode array detector (G7115A) (Agilent Technologies, Palo Alto, USA). Separation was performed on a Phenomenex Gemini C18 column (250 × 10 mm, 5 μm, 110 Å) with mobile phase A (water with 0.1% FA) and mobile phase B (ACN with 0.1% FA) at a flow rate of 4.0 mL/min with a 100 μL injection volume. Samples were dissolved in MeOH:water (4:6 *v/v*; 3 mL), filtered, and injected. The gradient program was 2–15% B (0–1.0 min), held at 15% B (1.0–15.0 min), then returned to 2% (15.0–16.0 min) for re-equilibration. Detection was at 254 and 280 nm. Peaks eluting at 7.0–7.7 (metabolite **3**), 8.6–9.5 (metabolite **2**), 9.6–10.7 (metabolite **1**), and 13.2–14.8 min (parent diCouSpd) were collected to afford pure diCouSpd and its metabolites **1**–**3** (Figure 1).

**Figure 1:**
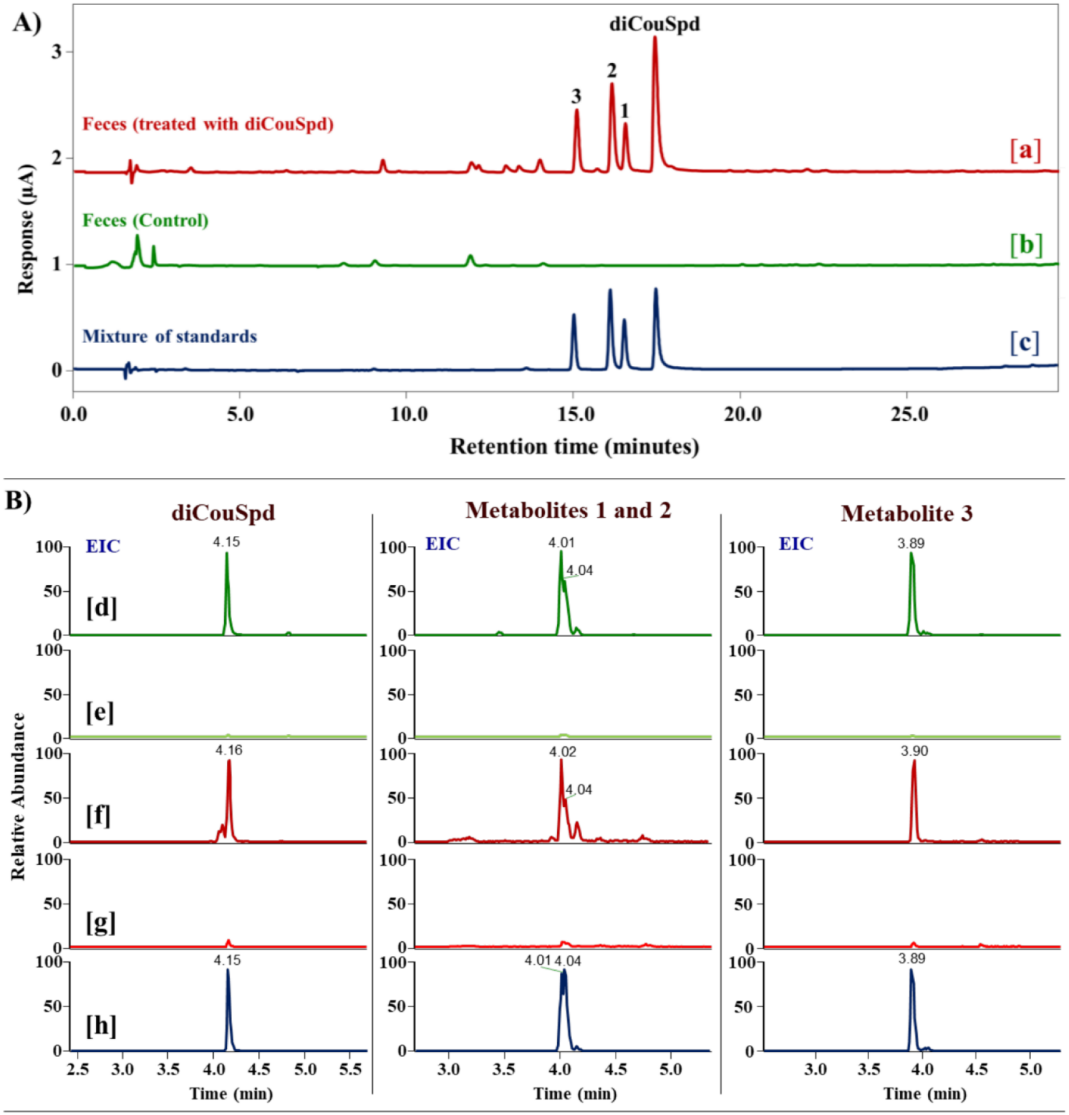
Detection of diCouSpd and its metabolites in mouse fecal and urine samples by HPLC–ECD and LC-MS. (**A**) HPLC–ECD chromatograms of diCouSpd and its metabolites: (**a**) fecal samples from mice treated with diCouSpd (200 mg/kg), (**b**) fecal samples from control mice, and (**c**) authentic diCouSpd and its metabolites. Data were acquired from the 400 mV channel. (**B**) LC-MS chromatograms of diCouSpd and its metabolites **1**–**3** filtered by the molecular ions (*m*/*z* 438.2, 440.2, and 442.2) in positive ion mode: (**d**) fecal samples from mice treated with diCouSpd (200 mg/kg), (**e**) fecal samples from control mice, (**f**) urine samples from treated mice, (**g**) urine samples from control mice, and (**h**) authentic diCouSpd and its synthesized metabolites. EIC: Extracted Ion Chromatogram.

Metabolite **1** [*N* ^1^-dihydro-*p*-coumaroyl-*N* ^10^-*p*-coumaroyl-spermidine (diHydroCou-Cou-Spd); Figures 2–4]: Light yellow viscous gum, (+)-HR-ESIMS *m*/*z* 440.2529 C_25_H_34_O_4_N_3_ [M+H]^+^ (calcd. for C_25_H_34_O_4_N_3_^+^, 440.2544). (−)-HR-ESIMS *m*/*z* 438.2402 C_25_H_32_O_4_N_3_ [M–H]^−^ (calcd. for C_25_H_32_O_4_N_3_^–^, 438.2398). ^1^H- and ^13^C NMR (400 and 100 MHz, in CD_3_OD) (Table 1 and Figures S1–S3).

**Figure 2:**
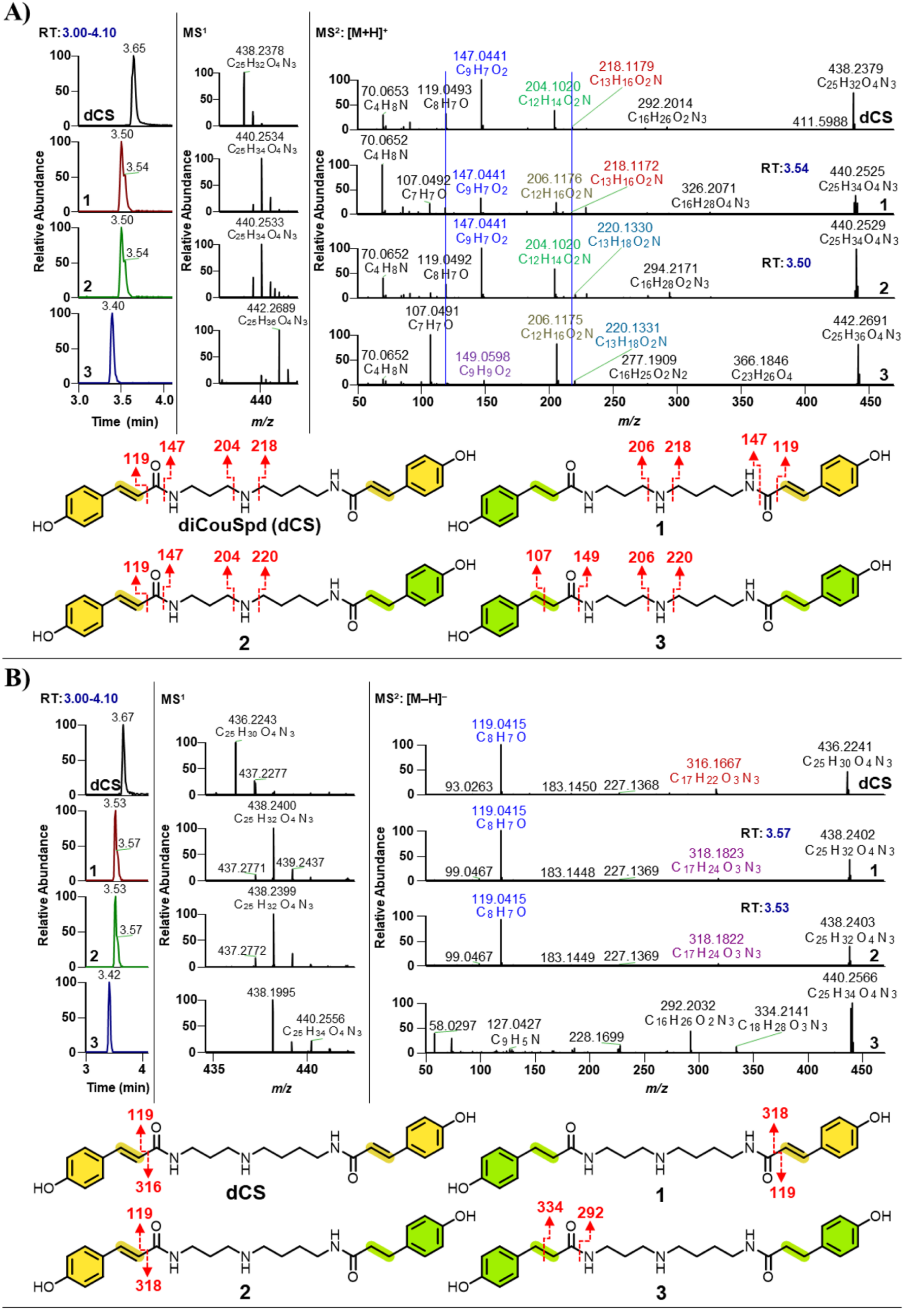
LC-MS characterization of diCouSpd (dCS) and metabolites **1–3** in positive (**A**) and negative (**B**) ion modes, including extracted ion chromatograms (EICs), MS^1^ spectra, MS/MS fragmentation spectra, and proposed fragmentation pathways. The data were acquired in a separate LC-MS run from Figure 1, resulting in a slight systematic retention time shift.

**Figure 3:**
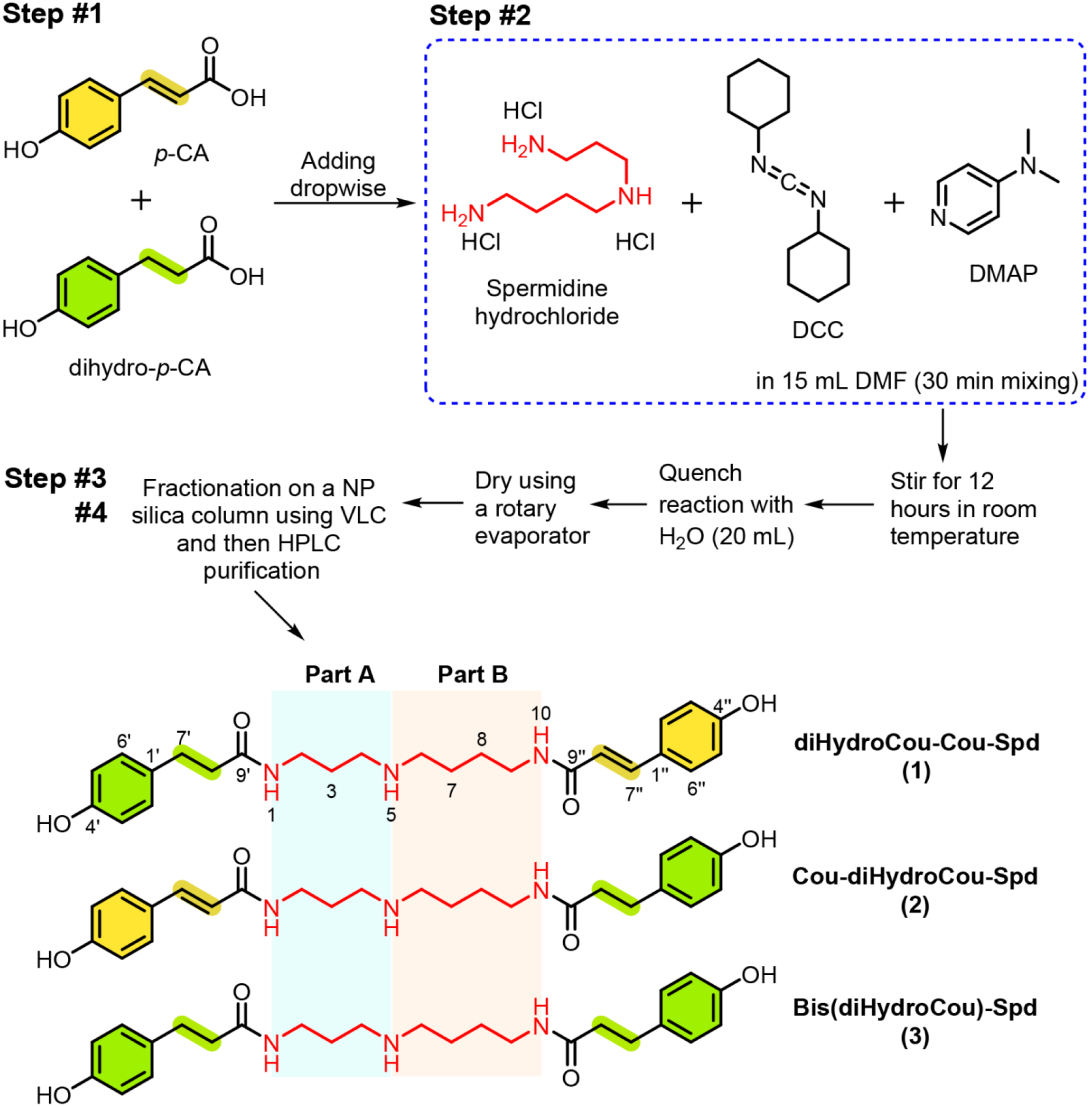
Synthesis procedure and chemical structures of diCouSpd metabolites **1**–**3**.

**Figure 4:**
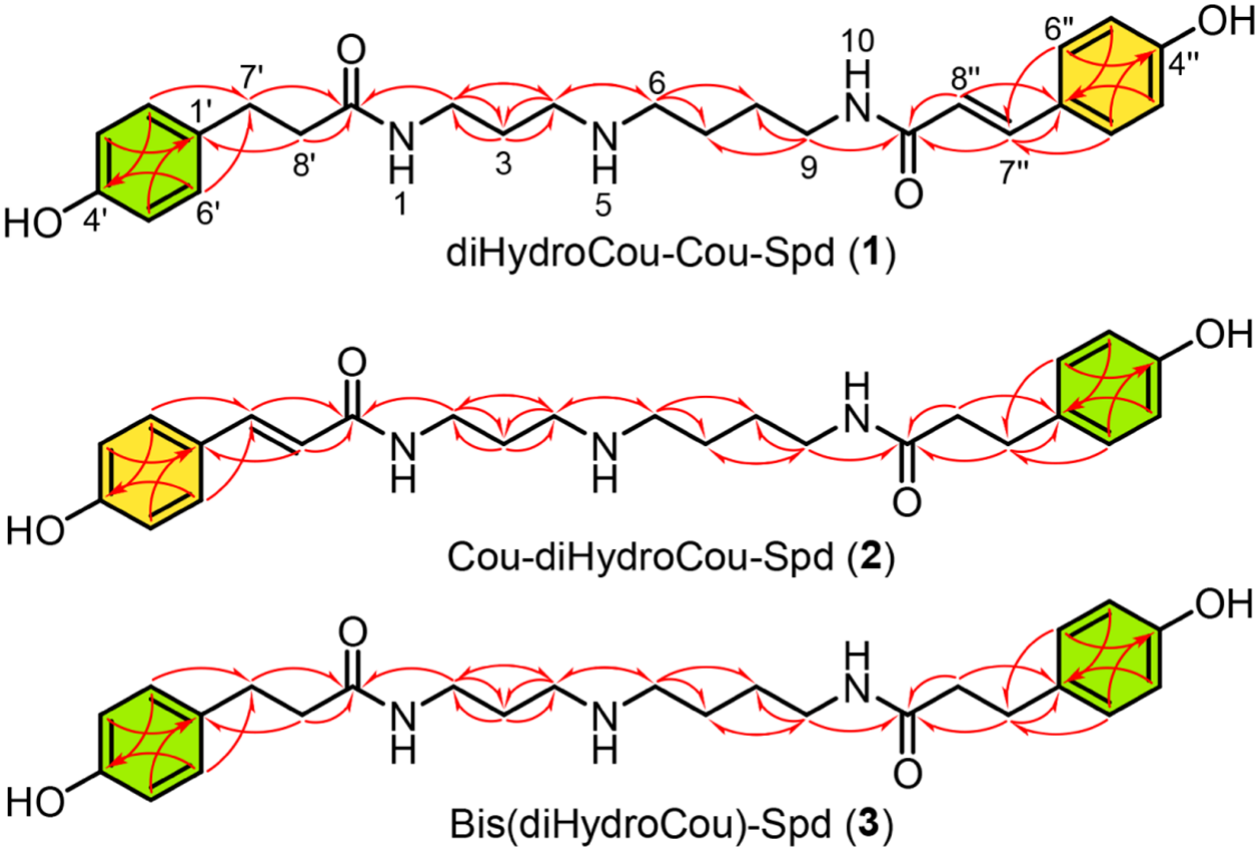
HMBC correlations of diCouSpd metabolites, including diHydroCou-Cou-Spd (**1**), Cou-diHydroCou-Spd (**2**), and Bis­(dihydroCou)-Spd (**3**).

**Table 1:** ^1^H and ^13^C NMR (400 and 100 MHz; CD_3_OD)^a^ of diCouSpd Metabolites, Including diHydroCou-Cou-Spd (**1**), Cou-diHydroCou-Spd (**2**), and Bis­(dihydroCou)-Spd (**3**)

	**Metabolite 1**		**Metabolite 2**		**Metabolite 3**	
No.	*δ*_H_ (mult, *J* in Hz)	*δ*_C_	*δ*_H_ (mult, *J* in Hz)	*δ*_C_	*δ*_H_ (mult, *J* in Hz)	*δ*_C_
**NH-1**	–	–	–	–	–	–
**2**	3.23 (dd, 1H, *J* = 7.0, 5.7)	36.6	3.42 (t, 1H, *J* = 6.5)	36.9	3.23 (dd, 2H, *J* = 7.0, 5.8)	36.6
**3**	1.75 (p, 1H, *J* = 7.0)	27.6	1.93 (p, 1H, *J* = 6.9)	27.9	1.75 (p, 2H, *J* = 7.0)	27.6
**4**	2.70 (t, 1H, *J* = 7.2)	46.0	3.00 (t, 1H, *J* = 6.4)	46.2	2.68 (t, 2H, *J* = 7.1)	46.0
**NH-5**	–	–	–	–	–	–
**6**	2.91 (dd, 1H, *J* = 8.0, 6.6)	48.7	2.97 (t, 1H, *J* = 6.4)	48.5	2.83 (m, 2H, overlapped)	48.9
**7**	1.66 (m, 2H)	24.6	1.52 (m, 2H)	27.5	1.51 (m, 2H)	27.5
**8**	1.69 (m, 2H)	27.7	1.60 (m, 2H)	24.5	1.55 (m, 2H)	24.5
**9**	3.36 (t, 1H, *J* = 6.6)	39.5	3.18 (t, 1H, *J* = 6.6)	39.26	3.17 (t, 2H, *J* = 6.4)	39.28
**NH-10**	–	–	–	–	–	–
**1′**	–	132.5	–	127.4	–	132.5
**2′**	7.04 (d, 1H, *J* = 8.5)	130.5	7.42 (d, 1H, *J* = 8.6)	130.4	7.04 (d, 1H, *J* = 8.6)	130.46
**3′**	6.71 (d, 1H, *J* = 8.5)	116.2	6.80 (d, 1H, *J* = 8.6)	116.8	6.71 (d, 1H, *J* = 8.6)	116.24
**4′**	–	156.9	–	160.9	–	156.9
**5′**	6.71 (d, 1H, *J* = 8.5)	116.2	6.80 (d, 1H, *J* = 8.6)	116.8	6.71 (d, 1H, *J* = 8.6)	116.24
**6′**	7.04 (d, 1H, *J* = 8.5)	130.5	7.42 (d, 1H, *J* = 8.6)	130.4	7.04 (d, 1H, *J* = 8.6)	130.46
**7′**	2.83 (t, 1H, *J* = 7.3)	31.8	7.51 (d, 1H, *J* = 15.7)	142.7	2.84 (t, 2H, *J* = 7.5)	31.8
**8′**	2.50 (t, 1H, *J* = 7.3)	38.6	6.42 (d, 1H, *J* = 15.7)	117.5	2.51 (t, 2H, *J* = 7.5)	38.6
**9′**	–	176.5	–	170.3	–	176.6
**4′–OH**	–	–	8.37 (brs)	–	–	–
**1″**	–	127.6	–	132.8	–	132.8
**2″**	7.41 (d, 1H, *J* = 8.5)	130.6	7.02 (d, 1H, *J* = 8.5)	130.7	7.02 (d, 1H, *J* = 8.6)	130.40
**3″**	6.80 (d, 1H, *J* = 8.5)	116.8	6.69 (d, 1H, *J* = 8.5)	116.2	6.69 (d, 1H, *J* = 8.6)	116.18
**4″**	–	160.7	–	156.8	–	156.8
**5″**	6.80 (d, 1H, *J* = 8.5)	116.8	6.69 (d, 1H, *J* = 8.5)	116.2	6.69 (d, 1H, *J* = 8.6)	116.18
**6″**	7.41 (d, 1H, *J* = 8.5)	130.6	7.02 (d, 1H, *J* = 8.5)	130.7	7.02 (d, 1H, *J* = 8.6)	130.40
**7″**	7.47 (d, 1H, *J* = 15.7)	142.1	2.81 (t, 1H, *J* = 7.55)	32.1	2.82 (t, 2H, *J* = 7.5)	32.2
**8″**	6.42 (d, 1H, *J* = 15.7)	118.1	2.43 (dd, 1H, *J* = 8.1, 7.0)	39.31	2.44 (dd, 2H, *J* = 8.1, 7.0)	39.32
**9″**	–	169.5	–	175.6	–	175.6
**4″–OH**	–	–	8.37 (brs)	–	–	–

a The assignments are based on ^1^H and ^13^C NMR broad bond (BB) and HMBC data.

Metabolite **2** [*N* ^1^-*p*-coumaroyl-*N* ^10^-dihydro-*p*-coumaroyl-spermidine (Cou-diHydroCou-Spd); Figures 2–4]: Light yellow viscous gum, (+)-HR-ESIMS *m*/*z* 440.2525 C_25_H_34_O_4_N_3_ [M+H]^+^ (calcd. for C_25_H_34_O_4_N_3_^+^, 440.2544). (−)-HR-ESIMS *m*/*z* 438.2403 C_25_H_32_O_4_N_3_ [M–H]^−^ (calcd. for C_25_H_32_O_4_N_3_^–^, 438.2398). ^1^H- and ^13^C NMR (400 and 100 MHz, in methanol-*d*_4_) (Table 1 and Figures S4–S6).

Metabolite **3** [*N* ^1^,*N* ^10^-bis­(dihydro-*p*-coumaroyl)-spermidine (Bis­(dihydroCou)-Spd); Figures 2–4]: Light yellow viscous gum, (+)-HR-ESIMS *m*/*z* 442.2691 C_25_H_36_O_4_N_3_ [M+H]^+^ (calcd. for C_25_H_36_O_4_N_3_^+^, 442.2700). (−)-HR-ESIMS *m*/*z* 440.2566 C_25_H_34_O_4_N_3_ [M–H]^−^ (calcd. for C_25_H_34_O_4_N_3_^–^, 440.2555). ^1^H- and ^13^C NMR (400 and 100 MHz, in CD_3_OD) (Table 1 and Figures S7–S9).

### 2.4 Nuclear Magnetic Resonance (NMR) Analysis

^1^H (400 MHz) and ^13^C (100 MHz) NMR spectra were acquired in deuterated methanol (CD_3_OD) on a 400 MHz NMR spectrometer (Bruker BioSpin, Rheinstetten, Germany). Chemical shifts (δ) are reported in ppm and coupling constants (*J*) in Hz.

### 2.5 Preparation of Corn Extract

One serving of WGs is defined as one ounce-equivalent (∼28 g grain ingredients) according to the USDA Dietary Guidelines (2020–2025) and Whole Grains Council standards. This typically corresponds to approximately 35–50 g of WG food products, depending on formulation and moisture content.^1,10^ In this study, 46 g was considered as one serving, and a commercial WG corn grits product purchased from Amazon was used. The extraction yield was approximately 10% (100 mg extract/g corn grits). Thus, two and four servings corresponded to 9.2 and 18.4 g of corn extract, respectively. Human equivalent doses were converted to mouse doses using body surface area normalization (*K*_m_ factors: human = 37, mouse = 3),^11^ resulting in gavage doses of 1.62 and 3.24 g extract/kg body weight, corresponding to two and four human servings, respectively.

The grits (200 g) were finely powdered using a SHARDOR Adjustable Coffee Grinder (Changsha Shardor Electrical Appliance Technology Co., Ltd., Changsha, China). The powdered material was extracted twice with 80% methanol (2 L per extraction) at ambient temperature under sonication and shaking for 1 h per cycle. The resulting dry extract was dissolved in 50% DMSO to prepare a stock solution (500 mg/mL) and stored at −20 °C until use for oral gavage administration.

### 2.6 Treatment of Mice and Sample Collection

#### 2.6.1 Animal Study and Sample Collection

Animal studies were conducted in accordance with the protocol approved by the Institutional Animal Care and Use Committee (IACUC) of the North Carolina Research Campus (No. 22-011). Male CD-1 mice (Charles River) were acclimated for ≥1 week prior to experimentation. Animals were housed (4 per cage) under controlled conditions (20 ± 2 °C, 50 ± 10% relative humidity, 12 h light/dark cycle, lights on 07:00–19:00) with ad libitum access to water and an AIN-93G diet.

***Experiment 1***: *High-dose administration for metabolite identification*

Mice were not fasted at any time during the experiment and had ad libitum access to food and water. DiCouSpd was administrated by oral gavage at 200 mg/kg body weight (prepared at 40 mg/mL in 50% DMSO). Immediately after dosing, animals (*n* = 4 per group) were transferred as a group into a single metabolic cage. Pooled urine and fecal samples (four mice per cage) were collected over a 24-h period. Control mice received vehicle (50% DMSO) under identical conditions. Both urine and fecal samples were immediately frozen and stored at −80 °C until analysis. This study was designed to facilitate metabolite discovery and structural elucidation of diCouSpd. Therefore, a relatively high dose was used to ensure sufficient exposure for metabolite identification.

***Experiment 2***: *Human intake-relevant exposure using WG corn extract*

The experimental procedures were identical to those described for *Experiment 1*, except those mice were administered WG corn extract corresponding to the estimated human intake of two or four servings of WG corn by oral gavage. Control mice received vehicle (50% DMSO) under identical conditions. This study was designed to access metabolite formation under human intake-relevant exposure conditions.

The use of 50% DMSO was required to ensure complete solubilization of diCouSpd and corn extract at their administered doses. In both experiments, control and treated mice were exposed to the same vehicle under identical conditions, allowing valid relative comparison of metabolic profiles.

#### 2.6.2 Mouse Sample Preparation

Biological samples (fecal and urine) were prepared as described previously with minor modifications.^12^ Briefly, freeze-dried fecal powder (50 mg) were homogenized in 80% MeOH (0.5 mL) containing 0.1% formic acid, sonicated for 30 min, and centrifuged (15000 × g, 4 °C, 15 min). Urine samples (200 μL) were mixed with NaOAc buffer (100 mM, pH 5, 340 μL), 10% ascorbic acid in NaOAc buffer (w/w, 12 μL), β-glucuronidase/sulfatase (48 μL), followed by incubation at 37 °C for 3 h. Proteins were precipitated by addition of ACN (1800 μL). The supernatant was dried under nitrogen and reconstituted in 100 μL 80% MeOH, vortexed, and centrifuged (15000 × g, 4 °C, 15 min) prior to LC-MS and HPLC-ECD analysis.

### 2.7 *In Vitro* Human Fecal Fermentation Study

Human fecal slurries (FS) were prepared from samples collected under a protocol approved by the Institutional Review Board (IRB) of North Carolina A&T State University (IRB# HS22-0041). Written informed consent was obtained from all participants prior to sample collection. Fecal samples were obtained from six healthy, nonsmoking participants (3 males, 3 females) who had not taken antibiotics for at least 6 months and were processed within 2 h under anaerobic conditions. Briefly, 50 g of feces was homogenized with 100 mL of 0.05% peptone water using a Seward stomacher for 3 min at 200 rpm. The homogenate was centrifuged (3000 × g, 4 °C, 2–3 min) to remove debris. The supernatant (fecal slurry) was mixed with sterile glycerol (final concentration 35%), aliquoted, and stored at −80 °C until use.^13^ Biotransformation of diCouSpd by pooled human FS was conducted following a previously reported protocol with minor modifications.^12^ Briefly, diCouSpd (50 μL, 10 mM in PBS), GAM medium (900 μL), and FS (100 μL) were mixed and aliquoted into seven sets (140 μL, each) corresponding to 0, 4, 8, 12, 24, 48, and 72 h. Two controls were included: (i) diCouSpd with GAM medium (control 1, no FS) to ensure metabolites did not arise from medium or incubation conditions alone; (ii) DMSO (50 μL), GAM medium (900 μL), and FS (100 μL) (control 2, no diCouSpd) to confirm metabolites were not endogenously produced by either microbiota or fecal matrix. Samples were incubated at 37 °C under anaerobic conditions and collected at designated time points. Each aliquot was quenched with an equal volume of MeOH, stored at −80 °C, then thawed, sonicated (10 min), and centrifuged (15000 × g, 4 °C, 15 min). Supernatants were analyzed by LC-MS and HPLC-ECD. In this study, pooled fecal slurry was used to maximize microbial diversity and facilitate metabolite discovery.

## 3 Results and Discussion

### 3.1 Metabolism of diCouSpd in Mice

To investigate metabolite discovery and structural elucidation, a single dose of diCouSpd (200 mg/kg) was orally administrated to CD-1 mice (*n* = 4), and biological samples (feces and urine) were collected over 24 h. Compared to the control group (*n* = 4), HPLC-ECD analysis of the fecal samples revealed three prominent peaks (metabolites **1**–**3**) (Figure 1A). Subsequent reevaluation of fecal and urine samples using LC-MS, alongside corresponding controls, confirmed the presence of these peaks in diCouSpd-treated urine, with *m*/*z* values of 440.2 (metabolites **1** and **2**) and 442.2 (metabolite **3**) (Figure 1B). Considering the mass differences relative to the parent compound (diCouSpd; *m*/*z* 438.2) and previously reported biotransformation of phenolamides, such as oat AVA-C (2c)^14^ and barley *p*-CouAgm,^12^ the observed increases of +2 Da (metabolites **1** and **2**) and +4 Da (metabolite **3**), suggest that these metabolites may arise from stepwise reduction of the double bonds.

### 3.2 UHPLC-HR-ESI-MS/MS Fragmentation Pattern of Metabolites **1**–**3**

To establish the structures of diCouSpd metabolites **1**–**3**, their MS/MS fragmentation patterns were analyzed using high-resolution mass spectrometry (HR-MS). Given that spermidine-derived phenolamides contain both nitrogen atoms (within the polyamine chain) and oxygen atoms (within the phenolic moieties), fragmentation behavior was investigated in both positive and negative ionization modes (Figure 2).

#### 3.2.1 MS/MS Fragmentation Pattern in Positive Ion Mode

The proposed fragmentation patterns and structural assignments were supported by previously reported studies.^15,16^ As an illustrative example, MS/MS analysis of diCouSpd ([M+H]^+^ at *m*/*z* 438.2379), acquired using higher-energy collisional dissociation (HCD, 40 eV), yielded major product ions at *m*/*z* 292, 218, 204, 147, 119, and 72 (Figure 2A), consistent with reported fragmentation of spermidine homoconjugates in barley.^15^ Detailed analysis revealed two key fragmentation features: (**a**) ions at *m*/*z* 204 (C_12_H_14_NO_2_) and 218 (C_13_H_16_NO_2_), indicating preferential cleavage at the *N* ^5^ position of the spermidine backbone [N^1^H_2_–(CH_2_)_3_–N^5^H–(CH_2_)_4_–N^10^H_2_], and (**b**) a characteristic *p*-coumaroyl ion at *m*/*z* 147 (C_9_H_7_O_2_). These fragments served as diagnostic markers for the characterization of diCouSpd and its metabolites. Two regioisomeric metabolites detected at 3.50 and 3.54 min (*m*/*z* 440.2533/440.2534, C_25_H_34_N_3_O_4_) exhibited distinct fragmentation patterns. One isomer produced fragment ions at *m*/*z* 204 and 220 (C_13_H_18_NO_2_), whereas the other showed ions at *m*/*z* 206 (C_12_H_16_NO_2_) and 218, indicating site-specific reduction occurring at the *p*-coumaroyl-diaminopropane (metabolite **1**) and the *p*-coumaroyl-putrescine (metabolite **2**) moieties, respectively (Figure 2A). Both isomers also generated a common fragment at *m*/*z* 294.2171 (C_16_H_28_N_3_O_2_), supporting reduction of the *p*-coumaroyl group. For metabolite **3** (3.40 min; *m*/*z* 442.2689), fragment ions at *m*/*z* 204 and 220 were observed, while the characteristic *p*-coumaroyl ion (*m*/*z* 147) was replaced by an ion at *m*/*z* 149.0598 (C_9_H_9_O_2_), confirming the presence of a dihydro-*p*-coumaroyl moiety (Figure 2A).

#### 3.2.2 MS/MS Fragmentation Pattern in Negative Ion Mode

In negative ion mode, diCouSpd and its metabolites **1**–**3** showed relatively simplified MS/MS fragmentation patterns, likely due to deprotonation of phenolic hydroxyl groups. As a representative example, MS/MS analysis of diCouSpd ([M–H]^−^ at *m*/*z* 436.2243), acquired using HCD at 40 eV, generated major product ions at *m*/*z* 316 (C_17_H_22_N_3_O_3_) and 119 (C_8_H_7_O), attributed to α-cleavage of the conjugated double bond-carbonyl system within the phenolic moieties (Figure 2B). Similar α-cleavage behavior in negative ion mode has been reported for isoliquiritigenin,^17^ caffeic acid,^18^ and *N* ^1^,*N* ^5^,*N* ^10^-tricoumaroyl-spermidine.^19^ These ions served as diagnostic markers for structural elucidation. Metabolites **1** and **2** displayed analogous fragmentation patterns, with characteristic ions at *m*/*z* 318 (C_17_H_24_N_3_O_3_) and 119, consistent with single double-bond reduction, occurring at different positions along the spermidine backbone. In contrast, metabolite **3** (*m*/*z* 440.2556, C_25_H_34_N_3_O_4_) did not display these diagnostic peaks. Instead, fragment ions at *m*/*z* 292.2032 (C_16_H_26_N_3_O_2_), corresponding to dihydro-*p*-coumaroyl spermidine, and *m*/*z* 334.2141 (C_18_H_28_N_3_O_3_) were observed, supporting reduction of both *p*-coumaroyl moieties.

### 3.3 Synthesis and NMR Elucidation of Metabolites **1**–**3**

To confirm the MS-based structural assignments, diCouSpd metabolites **1**–**3** were synthesized via amide bond formation using DCC/DMAP-mediated coupling (Figure 3). This approach enabled conjugation of *p*-coumaric acid (*p*-CA) and/or dihydro-*p*-coumaric acid (diH-*p*-CA) with spermidine trihydrochloride. For clarity in NMR structural interpretation, the spermidine backbone was divided into two segments: part A (diaminopropane) and part B (putrescine) (Figure 3).

#### 3.3.1 NMR Structural Elucidation of Metabolites **1** and **2**

The ^1^H NMR spectra of metabolites **1** and **2** showed similar aromatic proton signals in the δ_H_ 6.40–7.55 range, along with a characteristic *trans* olefinic system (δ_H_ 6.42 and 7.47, *J*_*7″,8″*_ = 15.7 Hz, for **1**; δ_H_ 6.42 and 7.51, *J*_*7′,8′*_ = 15.7 Hz, for **2**). The presence of additional methylene signals in the upfield (δ_H_ 2.40–2.85) supported reduction of one double bond in each compound. The ^13^C NMR spectra of both metabolites displayed 21 signals, fewer than the 25 carbons expected from the molecular formula C_25_H_34_N_3_O_4_ (Figure 2), indicating chemical equivalence within the aromatic moieties due to *para*-substitution-induced *pseudo*-symmetry of aromatic carbons. Significant chemical shift differences (Δδ_C_ > 6 ppm) in the amide carbonyl carbons (δ_C_ 176.5 and 169.5 for C-9′ and C-9″ in **1**; δ_C_ 170.3 and 175.6 for C-9′ and C-9″ in **2**) further supported structural variation between the two regioisomers (Table 1; Figures S2 and S5). Key diagnostic differences in the spermidine backbone clearly distinguished the two isomers (Table 1; Figures S1 and S4). For metabolite **1**, downfield/upfield patterns for H_2_-2–H_2_-4 (part A; δ_H_ 3.23, 1.75, 2.70) indicated reduction adjacent to the diaminopropane unit [dihydro-*p*-coumaroyl–N^1^H_2_–(CH_2_)_3_–N^5^H−], while H_2_-9 (δ_H_ 3.36) supported *p*-coumaroyl substitution on part B [−N^5^H–(CH_2_)_4_–N^10^H–*p*-coumaroyl] (Table 1; Figure S1). For metabolite **2**, opposite chemical shifts trends (H_2_-2–H_2_-4 at δ_H_ 3.42, 1.93, 3.00; H_2_-9 at δ_H_ 3.18), indicated reversed substitution pattern (Table 1; Figure S4). These assignments were further corroborated by HMBC correlations. For metabolite **1**, correlations from H_2_-2 to C-9′ (δ_C_ 176.5)/C-4 (δ_C_ 46.0), from H_2_-7′ (δ_H_ 2.83)/H_2_-8′ (δ_H_ 2.50) to C-9′, from H_2_-9 (δ_H_ 3.36) to C-9″ (δ_C_ 169.5), and from H-7″ (δ_H_ 7.47)/H_2_-8″ (δ_H_ 6.42) to C-9″ (Figures 4, S3) established that the dihydro-*p*-coumaroyl moiety is attached to the aminopropane segment, while the *p*-coumaroyl moiety is linked to the putrescine segment. For metabolite **2**, HMBC correlations from H_2_-2 to C-9′ (δ_C_ 170.3)/C-4 (δ_C_ 46.2), from H_2_-7′ (δ_H_ 7.51)/H_2_-8′ (δ_H_ 7.42) to C-9′, from H_2_-9 (δ_H_ 3.18) to C-9″ (δ_C_ 175.6), and from H-7″ (δ_H_ 2.81)/H_2_-8″ (δ_H_ 2.43) to C-9″ (Figures 4, S6) confirmed the opposite substitution pattern, with the dihydro-*p*-coumaroyl moiety attached to the putrescine segment and the *p*-coumaroyl moiety linked to the aminopropane segment. Accordingly, these metabolites designated as *N* ^1^-dihydro-*p*-coumaroyl-*N* ^10^-*p*-coumaroyl-spermidine [**1**; diHydroCou-Cou-Spd] and *N* ^1^-*p*-coumaroyl-*N* ^10^-dihydro-*p*-coumaroyl-spermidine [**2**; Cou-diHydroCou-Spd]. Previously reported reduced spermidine-derived phenolamides in black goji berry (*N* ^1^-dihydrocaffeoyl-*N* ^10^-caffeoyl-spermidine)^20^ and in wolfberry (*N* ^1^-caffeoyl-*N* ^10^-dihydrocaffeoyl-spermidine)^21^ support the plausibility of such partially hydrogenated derivatives, although metabolites **1** and **2** have not, to the best of our knowledge, been previously reported.

#### 3.3.2 NMR Structural Elucidation of Metabolite **3**

The ^1^H NMR spectrum showed simplified aromatic signals at δ_H_ 7.04 (d, *J* = 8.6 Hz, H-2′/H-6′) and 6.71 (d, *J* = 8.6 Hz, H-3′/H-5′), as well as δ_H_ 7.02 (d, *J* = 8.6 Hz, H-2″/H-6″) and 6.69 (d, *J* = 8.6 Hz, H-3″/H-5″), consistent with *para*-substituted phenyl rings and analogous to the patterns observed for metabolites **1** and **2** (Table 1; Figure S7). Consistent with this assignment, the ^13^C NMR spectrum displayed 21 signals, fewer than the 25 carbons expected for C_25_H_36_N_3_O_4_ (Figure 2), indicating chemical equivalence within the aromatic moieties due to *para*-substitution-induced *pseudo*-symmetry of aromatic carbons. The close similarity in chemical shifts among corresponding carbons further supports a *pseudo*-symmetrical structure arising from nonequivalent substitution along the spermidine backbone (parts A and B) (Figure 3). Amide carbonyl signals at δ_C_ 176.6 (C-9′) and 175.6 (C-9″) are consistent with saturation of both side-chain double bonds relative to diCouSpd (Table 1; Figure S8). Key HMBC correlations further confirmed the structure: for part A, correlations from H_2_-2 (δ_H_ 3.23) to C-9′ and C-4 (δ_C_ 46.0), and from H_2_-7′ (δ_H_ 2.84)/H_2_-8′ (δ_H_ 2.51) to C-9′; For part B, correlations from H_2_-9 (δ_H_ 3.17) to C-9″, and from H-7″ (δ_H_ 2.82)/H_2_-8″ (δ_H_ 2.44) to C-9″ (Figures 4 and S9). Although reduced spermidine-derived phenolamides, such as *N* ^1^,*N* ^10^-bis­(dihydrocaffeoyl)-spermidine, have been reported in potato tubers^22^ and in lulo (*Solanum quitoense* Lam.),^23^ the structure of metabolite **3** has never been previously described. Accordingly, it is herein identified as *N* ^1^,*N* ^10^-bis­(dihydro-*p*-coumaroyl)-spermidine [**3**; bis­(diHydroCou)-Spd].

### 3.4 Confirmation of diCouSpd Metabolite Formation Following Dietary-Relevant Corn Intake

To further evaluate the physiological relevance of the identified metabolites, an additional mouse experiment was conducted using corn extract at doses corresponding to two and four human servings of WG corn. Analysis of 24 h fecal and urine samples confirmed the formation of all three reduced metabolites following oral administration of corn extract (Figure 5). At the two-serving dose, metabolites **1**–**3** were readily detected in fecal samples, with metabolite **3** appearing to be the major detectable metabolite, whereas the parent compound diCouSpd was present at comparatively lower levels. In contrast, urine samples were dominated by the intact diCouSpd, while metabolites **1**–**3** were present only at trace or undetectable levels. The detection of metabolites **1**–**3** predominantly in fecal samples is consistent with microbial transformation of diCouSpd occurring in the intestinal lumen. However, because the present study was designed for qualitative metabolite identification rather than quantitative pharmacokinetic analysis, the absence or low abundance of metabolites **1**–**3** in urine should not be interpreted as evidence of limited intestinal absorption. Additional pharmacokinetic studies with an extended urine collection period beyond 24 h are required to determine whether these metabolites are absorbed and subsequently excreted at later time points. At the four-serving dose, metabolites **1** and **2** appeared relatively more prominent than metabolite **3** in the representative fecal chromatograms. However, these chromatograms are intended for qualitative illustration only, and no conclusions regarding dose-dependent metabolite formation or relative metabolite abundance should be drawn from these data. These results demonstrate that the reduced metabolites identified in the high-dose metabolite discovery experiment are also formed following dietary-relevant exposure to WG corn. The identities of diCouSpd and metabolites **1–3** were further confirmed by MS/MS fragmentation analysis (Figure 5C), which was consistent with the fragmentation patterns described in Section 3.2.

**Figure 5:**
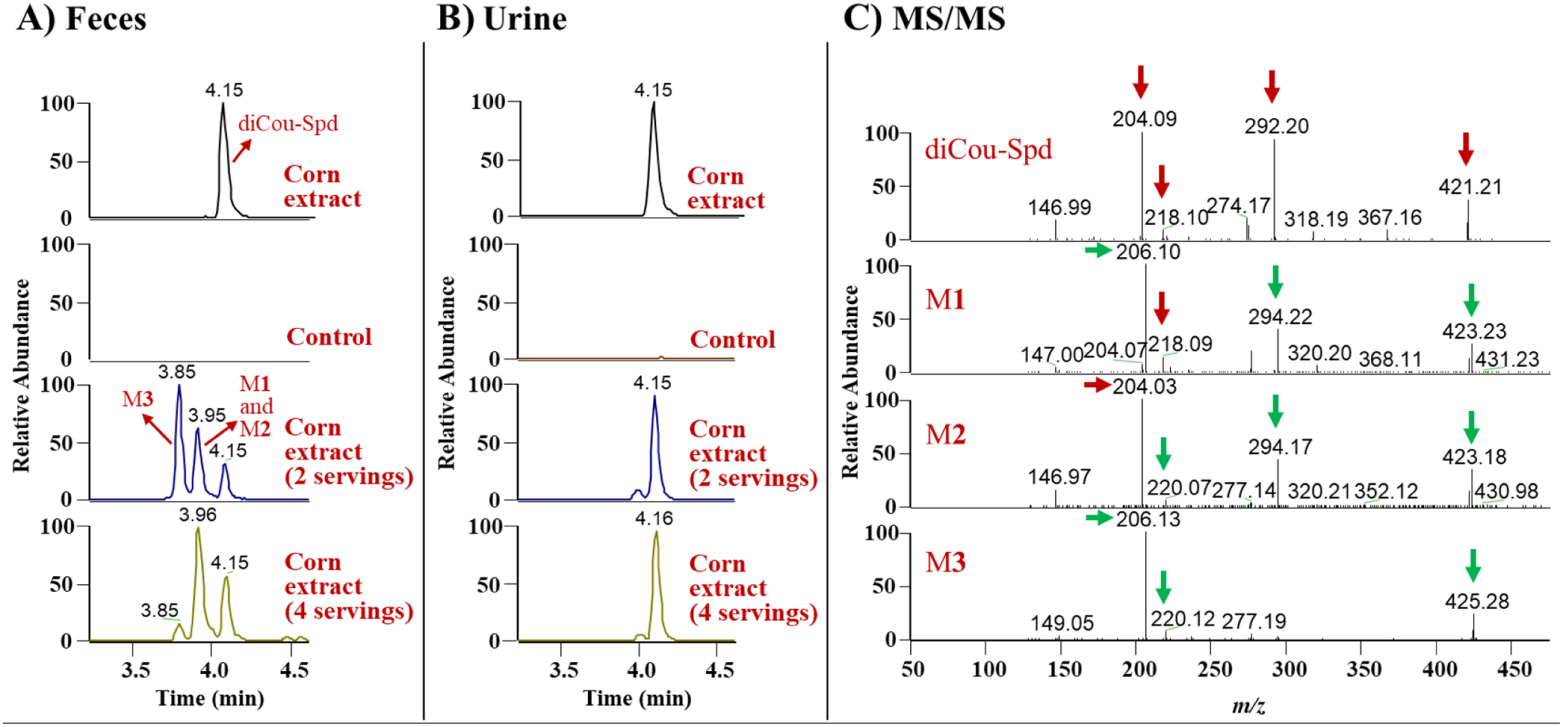
LC-MS spectra and MS/MS fragmentation patterns of diCou-Spd and its metabolites **1**–**3**. **A**) Compounds detected in mouse feces following administration of whole-grain corn grits extract equivalent to two and four servings; **B**) Compounds detected in urine under the same dosing conditions; **C**) MS/MS fragmentation patterns of diCou-Spd and its metabolites **1**–**3** acquired in positive ion mode on an LTQ ion trap mass spectrometer. The predominant precursor ion observed under these conditions was [M+H–NH_3_]^+^, which was selected for MS/MS analysis. Red arrows indicate intact fragment ion in parent diCou-Spd, while green arrows donate the reduced portions of the molecule.

### 3.5 Metabolism of diCouSpd by Human Gut Microbiota

To investigate the microbial biotransformation of diCouSpd, the compound was incubated under anaerobic conditions with a pooled human fecal slurry obtained from six healthy adults. Samples collected at 0, 4, 8, 12, 24, 48, and 72 h were analyzed by HPLC-ECD (Figure 6) and compared with authentic synthesized standards. After 12 h of incubation, diCouSpd was partially converted to metabolites **1** (diHydroCou-Cou-Spd) and **2** (Cou-diHydroCou-Spd). With extended incubation (≥24 h), the parent compound was completely depleted, and intermediates **1** and **2** were associated with the appearance and accumulation of metabolite **3** [bis­(diHydroCou)-Spd]. This temporal distribution of metabolites supports a putative stepwise hydrogenation pathway under anaerobic microbial conditions, in which sequential reduction of the *p*-coumaroyl leads to the corresponding dihydro derivatives. The accumulation of bis­(diHydroCou)-Spd (**3**) at later time points indicates that it represents the terminal product of this biotransformation pathway. Control experiments confirmed the microbiota-dependent nature of these transformations. In the absence of fecal slurry (control 1), diCouSpd remained stable over 72 h, excluding nonbiological hydrogenation or chemical degradation. Conversely, incubation of fecal slurry without diCouSpd (control 2) produced no detectable metabolites, confirming that these products are not endogenously generated by the microbiota (Figure 6). It is worth noting that these findings reflect metabolic capacity under pooled microbiota conditions and do not reflect interindividual variability in gut microbial composition.

**Figure 6:**
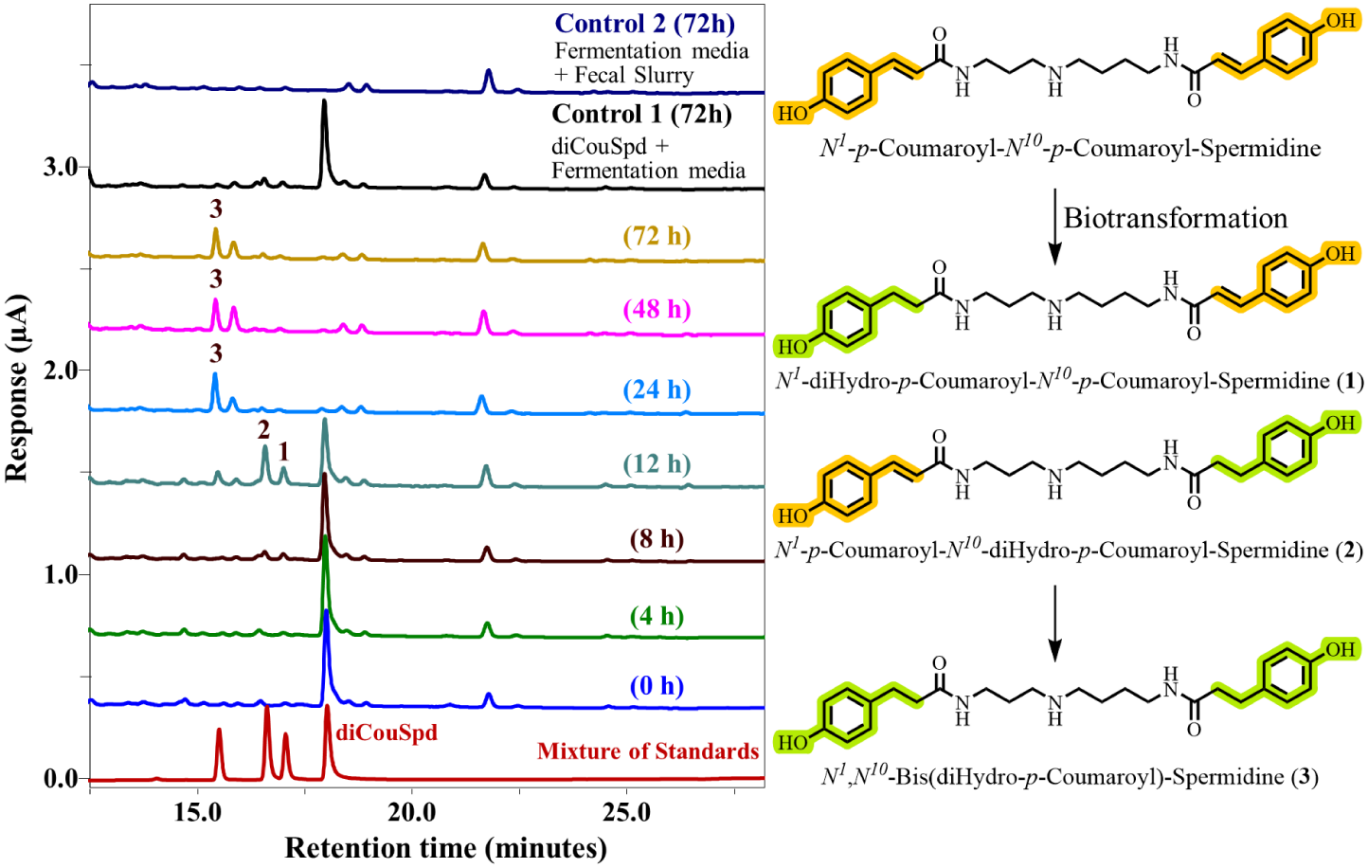
HPLC–ECD chromatograms of diCouSpd incubated with human fecal slurry at different time points (0, 4, 8, 12, 24, 48, and 72 h), together with control 1 (to confirm that the metabolites were not produced by time-dependent transformation of diCouSpd in the fermentation medium), control 2 (to confirm that the metabolites were not endogenously derived from the gut microbiota), and authentic diCouSpd and its synthesized metabolites **1**–**3**. Data were acquired from the 400 mV channel.

Natural products, including stilbenoids, flavonoids, chalcones, lignans, hydroxycinnamic acids, steroids, and phenolamides (i.e., oat AVA-C and barley *p*-CouAgm), commonly contain conjugated double bonds that represent key targets for microbial biotransformation, particularly reductive hydrogenation by the gut microbiota.^12,14,24^ Although studies specially addressing phenolamide metabolism remain limited, available evidence supports microbiota-driven reduction pathways. For instance, oat AVA-C is converted to its dihydro derivative by human and murine gut microbiota, with the reduced metabolite exhibiting enhanced cytotoxicity against human colon cancer cells compared to the parent compound.^14^ Similarly, barley *p*-CouAgm undergoes microbial reduction to its corresponding dihydro form. However, the extent of transformation varies across individuals, reflecting interindividual differences in gut microbial composition rather than a single defined biochemical pathway.^12^

This study elucidates the biotransformation of diCouSpd, a major spermidine-derived phenolamide in corn, using integrated structural characterization together with *in vivo* mouse and *in vitro* human fecal fermentation models. Three previously unreported metabolites were identified as regioisomeric and hydrogenated derivatives: *N* ^*1*^-dihydro-*p*-coumaroyl-*N* ^*10*^-*p*-coumaroyl-spermidine [**1**; diHydroCou-Cou-Spd], *N* ^*1*^-*p*-coumaroyl-*N* ^*10*^-dihydro-*p*-coumaroyl-spermidine [**2**; Cou-diHydroCou-Spd], and *N* ^*1*^,*N* ^*10*^-bis­(dihydro-*p*-coumaroyl)-spermidine [**3**; bis­(diHydroCou)-Spd]. To our knowledge, these metabolites have not been previously reported *in vivo*. Importantly, all three metabolites were also detected following oral administration of WG corn extract at dietary-relevant intake levels (equivalent to two and four human servings), demonstrating that they are formed under physiologically relevant exposure conditions. The time-dependent formation of these metabolites in human fecal fermentation models supports the involvement of gut microbiota in the hydrogenation of diCouSpd. Given the biological importance of spermidine and the known effects of double-bond reduction on molecular polarity and bioactivity, these structural modifications may influence the bioavailability, biological functions, and host-microbiome interactions of corn-derived phenolamides. However, further studies are required to determine their bioavailability and functional relevance *in vivo*. Overall, this study identifies diCouSpd and its reduced metabolites as potential exposure biomarkers of WG corn intake and provides new insights into the gut microbial reductive metabolism of dietary phenolamides. Further controlled human feeding studies are warranted to validate their utility as biomarkers of corn consumption.

## Supplementary Material



## Notes

^1^H NMR (400 MHz, CD_3_OD), ^13^C NMR (100 MHz, CD_3_OD), and HMBC spectra of synthesized *N* ^1^-dihydro-*p*-coumaroyl-*N* ^10^-*p*-coumaroyl-spermidine (metabolite **1**) (Figures S1–S3), *N* ^1^-*p*-coumaroyl-*N* ^10^-dihydro-*p*-coumaroyl-spermidine (metabolite **2**) (Figures S4–S6), and *N* ^1^,*N* ^10^-bis­(dihydro-*p*-coumaroyl)-spermidine (metabolite **3**) (Figures S7–S9) (PDF)
