# Supplementary material for: Biotransformation of Corn-Derived N 1,N 10-di‑p‑Coumaroyl Spermidine in Mice and by Human Gut Microbiota Reveals Novel Reduced Metabolites
Source: J Agric Food Chem. 2026 Aug 3;74(31):24426–36. doi: 10.1021/acs.jafc.6c06580 (PMC13474957; doi:10.1021/acs.jafc.6c06580)
Supplement: Supplementary file 1 [file jf6c06580_si_001.pdf]

Supporting Information

Biotransformation of Corn-Derived *N*^{1},*N*^{10}-di-*p*-Coumaroyl Spermidine in Mice and by Human Gut Microbiota Reveals Novel Reduced Metabolites

Salar Hafez-Ghoran[1], Nathaniel Yeboah[2], Shengmin Sang[1]*

[1.] *Laboratory for Functional Foods and Human Health, Center for Excellence in Post-Harvest Technologies, North Carolina Agricultural and Technical State University, North Carolina Research Campus, 500 Laureate Way, Kannapolis, North Carolina 28081, United States.*

[2.] *Department of Chemistry, North Carolina Agricultural and Technical State University, Greensboro, North Carolina 27411, United States.*

***Correspondence:**

Shengmin Sang

Tel, 704-250-5710

Email address: ssang@ncat.edu

Contents

Contents		Page
**Figure S1**.	^{1}H NMR (400 MHz, CD_3OD) spectrum of synthesized N^{1}-dihydro-*p*-coumaroyl-N^{10}-*p*-coumaroyl-spermidine (metabolite **1**).	S3
**Figure S2**.	^{13}C NMR (100 MHz, CD_3OD) spectrum of synthesized N^{1}-dihydro-*p*-coumaroyl-N^{10}-*p*-coumaroyl-spermidine (metabolite **1**).	S3
**Figure S3**.	HMBC spectrum of synthesized N^{1}-dihydro-*p*-coumaroyl-N^{10}-*p*-coumaroyl-spermidine (metabolite **1**).	S4
**Figure S4**.	^{1}H NMR (400 MHz, CD_3OD) spectrum of synthesized N^{1}-*p*-coumaroyl-N^{10}-dihydro-*p*-coumaroyl-spermidine (metabolite **2**).	S4
**Figure S5**.	^{13}C NMR (100 MHz, CD_3OD) spectrum of synthesized N^{1}-*p*-coumaroyl-N^{10}-dihydro-*p*-coumaroyl-spermidine (metabolite **2**).	S5
**Figure S6**.	HMBC spectrum of synthesized N^{1}-*p*-coumaroyl-N^{10}-dihydro-*p*-coumaroyl-spermidine (metabolite **2**).	S5
**Figure S7**.	^{1}H NMR (400 MHz, CD_3OD) spectrum of synthesized N^{1},N^{10}-bis(dihydro-*p*-coumaroyl)-spermidine (metabolite **3**).	S6
**Figure S8**.	^{13}C NMR (100 MHz, CD_3OD) spectrum of synthesized N^{1},N^{10}-bis(dihydro-*p*-coumaroyl)-spermidine (metabolite **3**).	S6
**Figure S9**.	HMBC spectrum of synthesized N^{1},N^{10}-bis(dihydro-*p*-coumaroyl)-spermidine (metabolite **3**).	S7

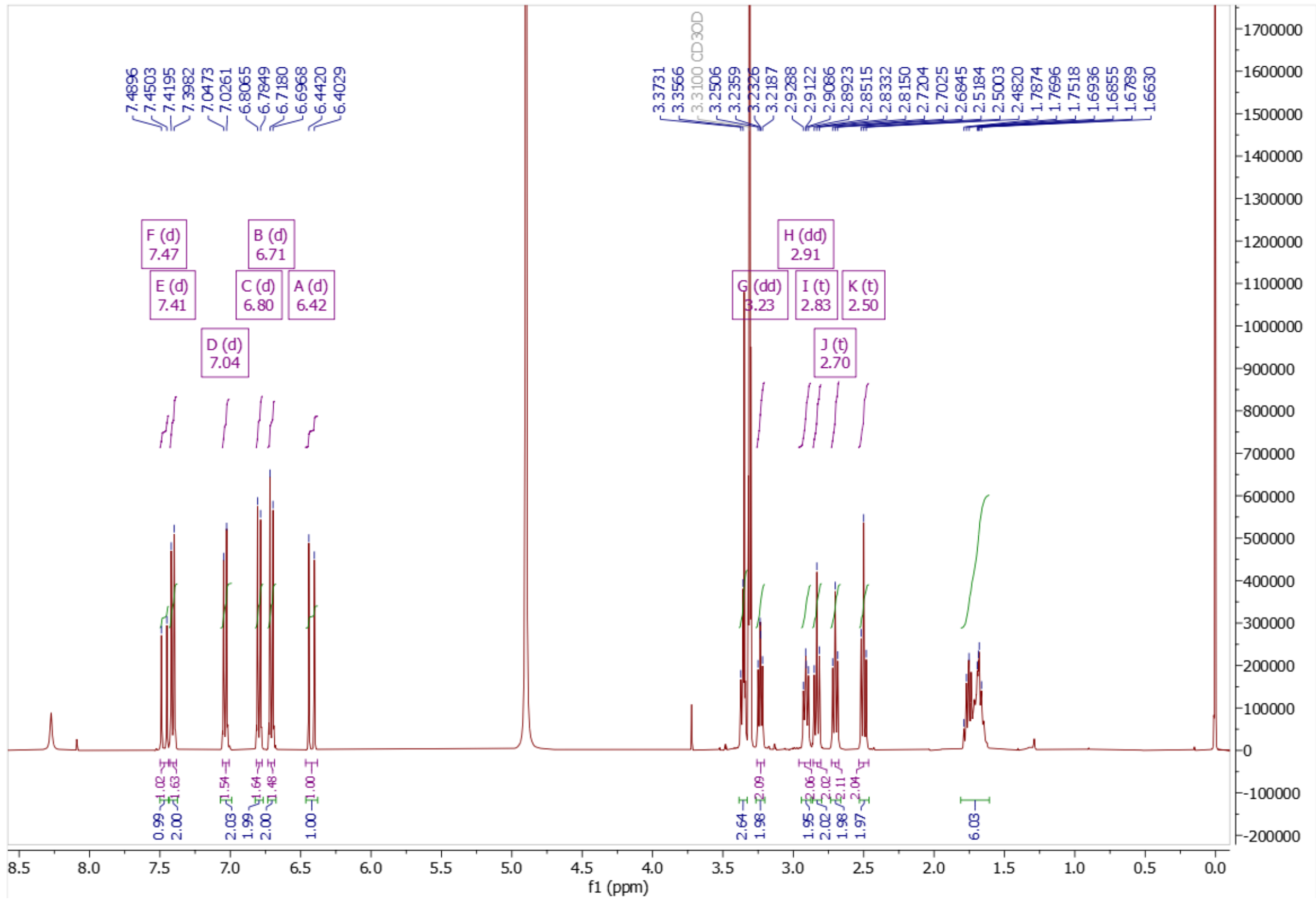

Figure S1. ^{1}H NMR (400 MHz, CD_3OD) spectrum of synthesized *N*[1]-dihydro-*p*-coumaroyl-*N*[10]-*p*-coumaroyl-spermidine (metabolite **1**).

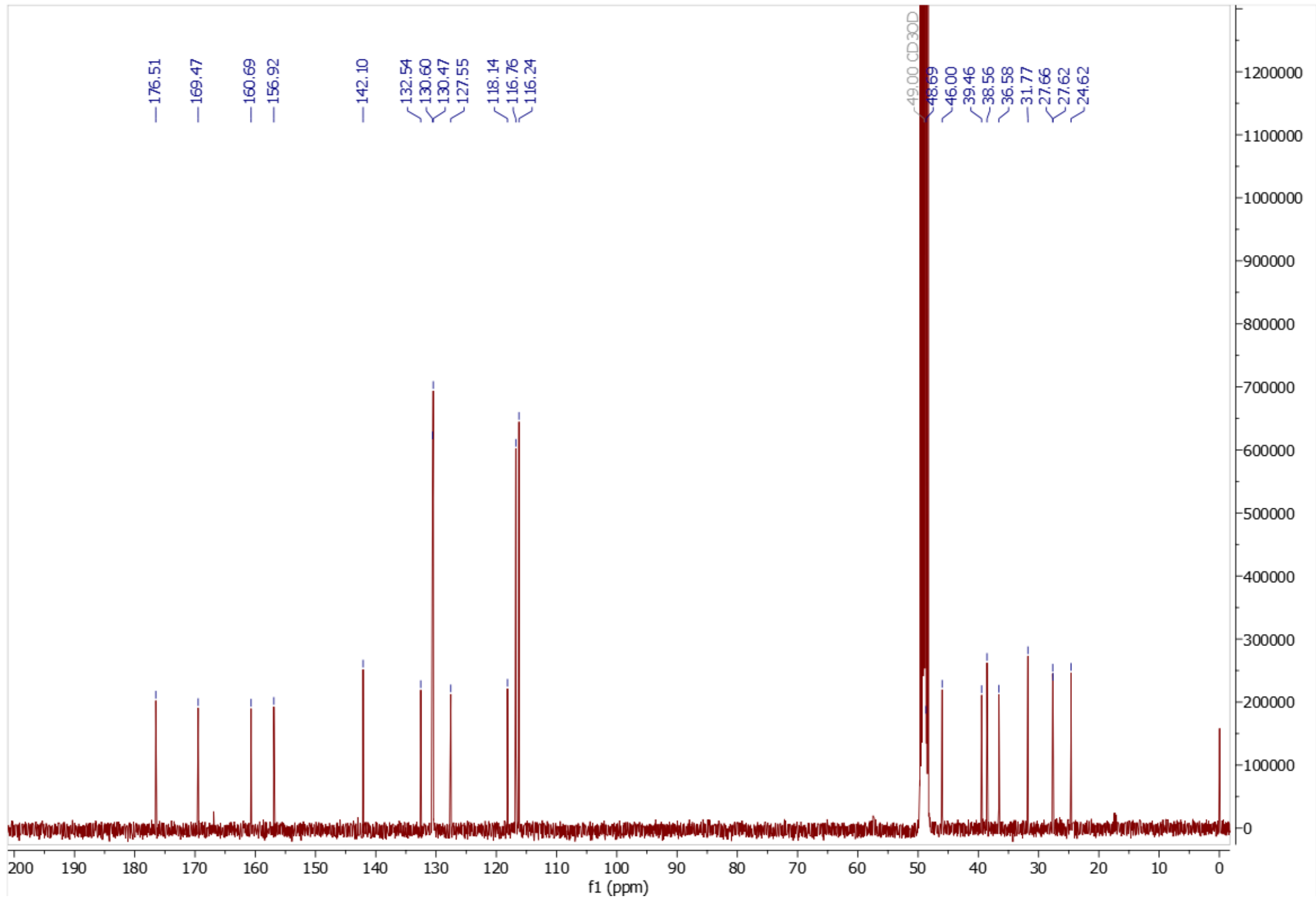

Figure S2. ^{13}C NMR (100 MHz, CD_3OD) spectrum of synthesized *N*[1]-dihydro-*p*-coumaroyl-*N*[10]-*p*-coumaroyl-spermidine (metabolite **1**).

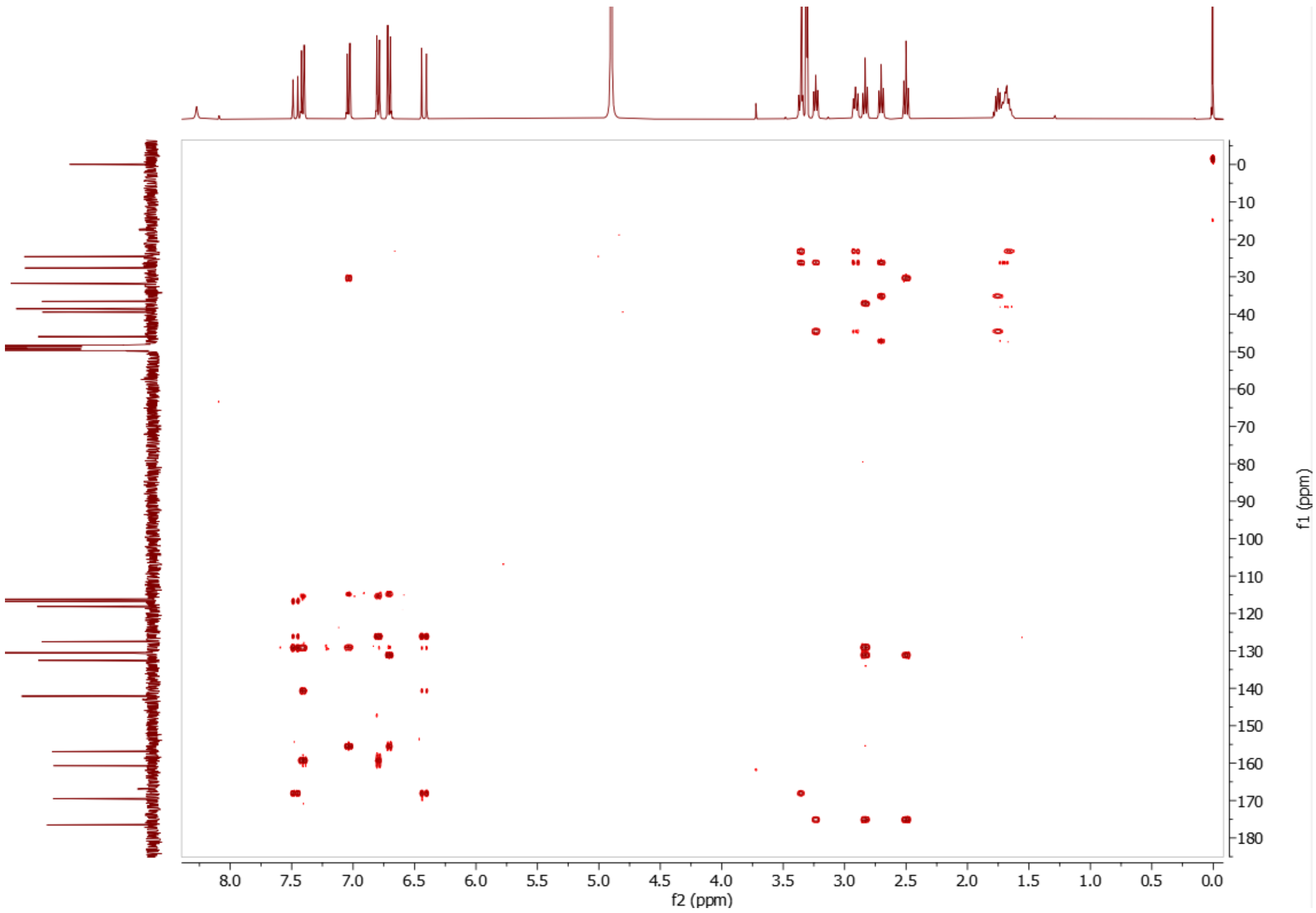

Figure S3. HMBC spectrum of synthesized *N*[1]-dihydro-*p*-coumaroyl-*N*[10]-*p*-coumaroyl-spermidine (metabolite **1**).

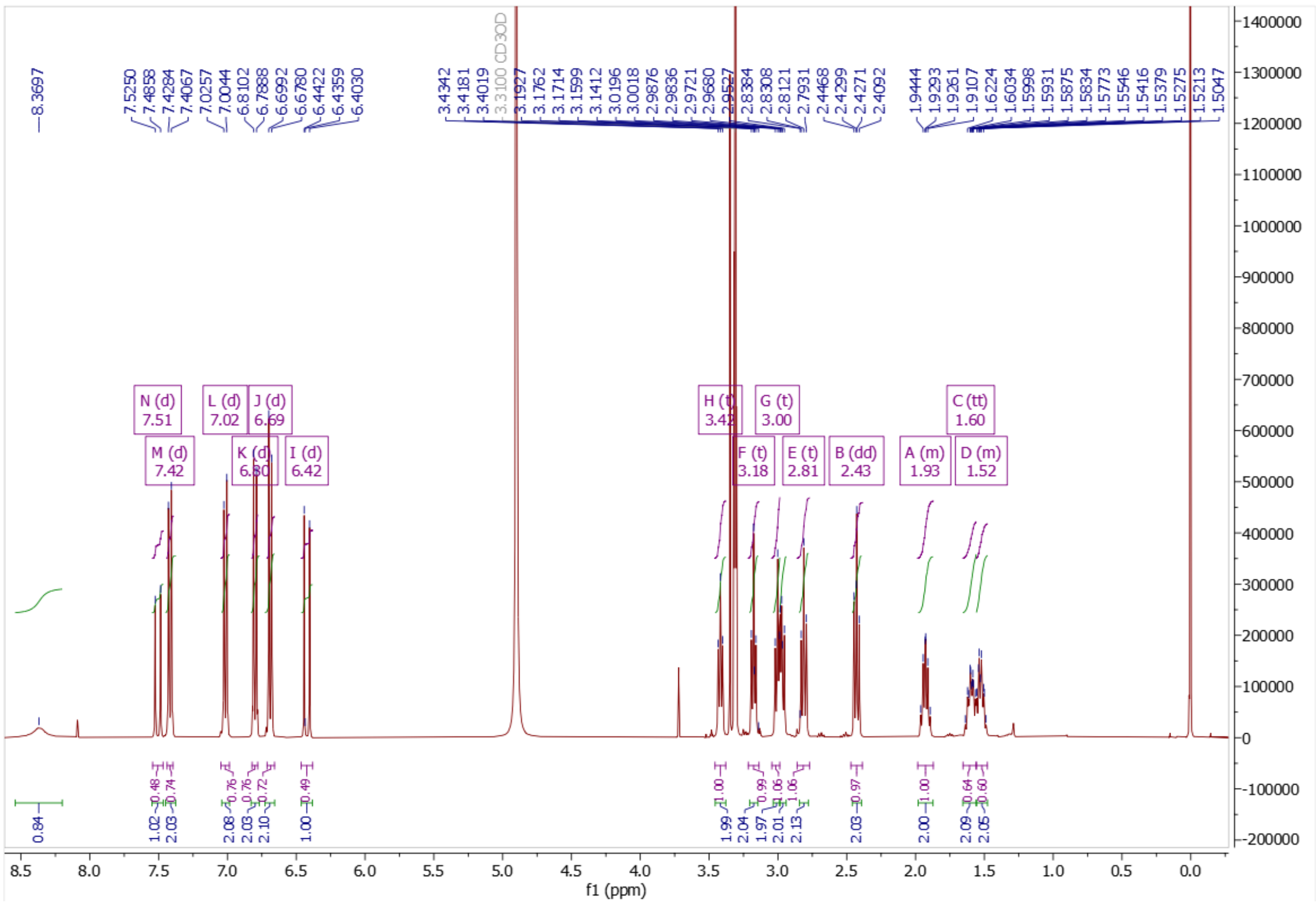

Figure S4. ^{1}H NMR (400 MHz, CD_3OD) spectrum of synthesized *N*[1]-*p*-coumaroyl-*N*[10]-dihydro-*p*-coumaroyl-spermidine (metabolite **2**).

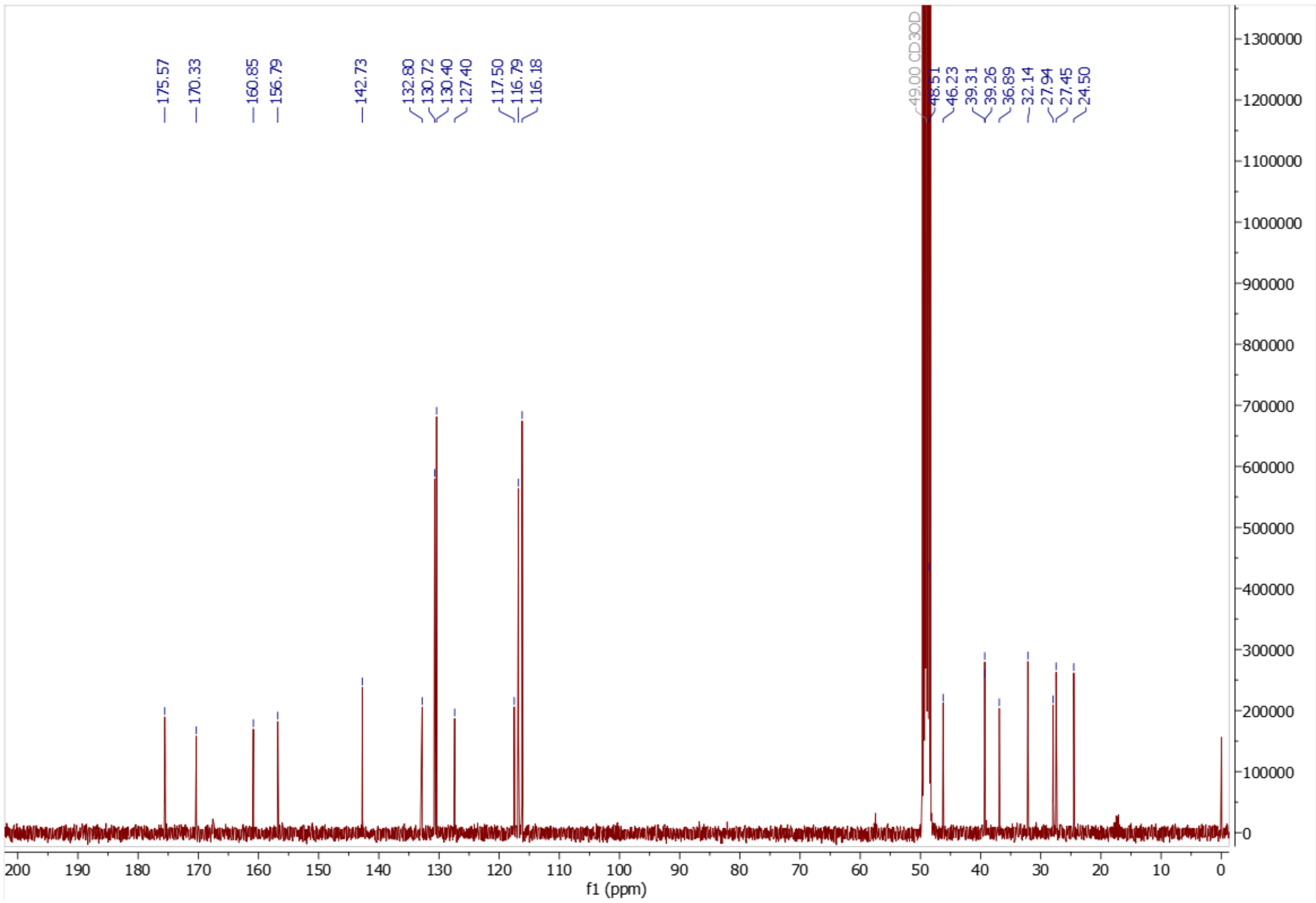

Figure S5. ^{13}C NMR (100 MHz, CD_3OD) spectrum of synthesized *N*[1]-*p*-coumaroyl-*N*[10]-dihydro-*p*-coumaroyl-spermidine (metabolite **2**).

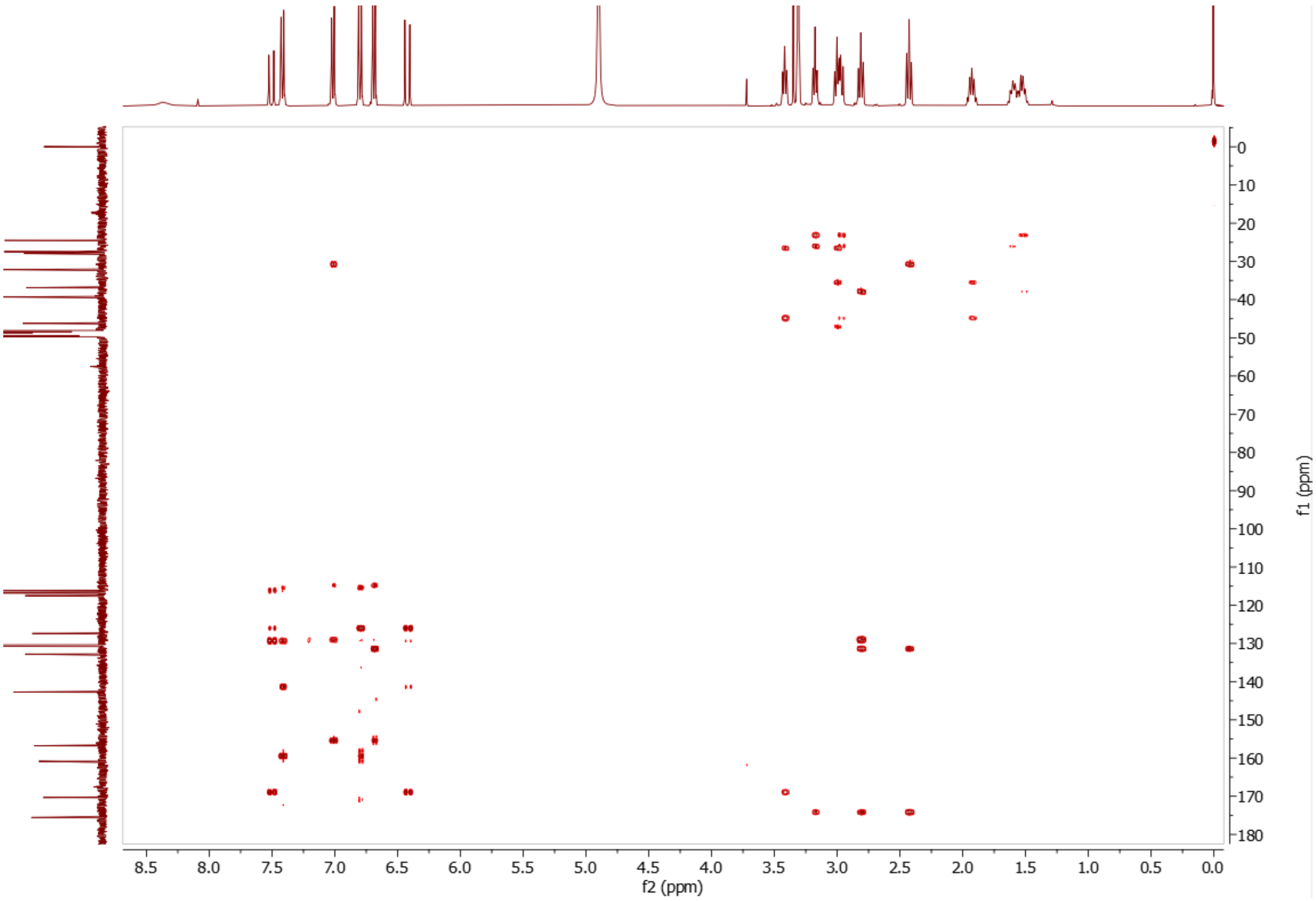

Figure S6. HMBC spectrum of synthesized *N*[1]-*p*-coumaroyl-*N*[10]-dihydro-*p*-coumaroyl-spermidine (metabolite **2**).

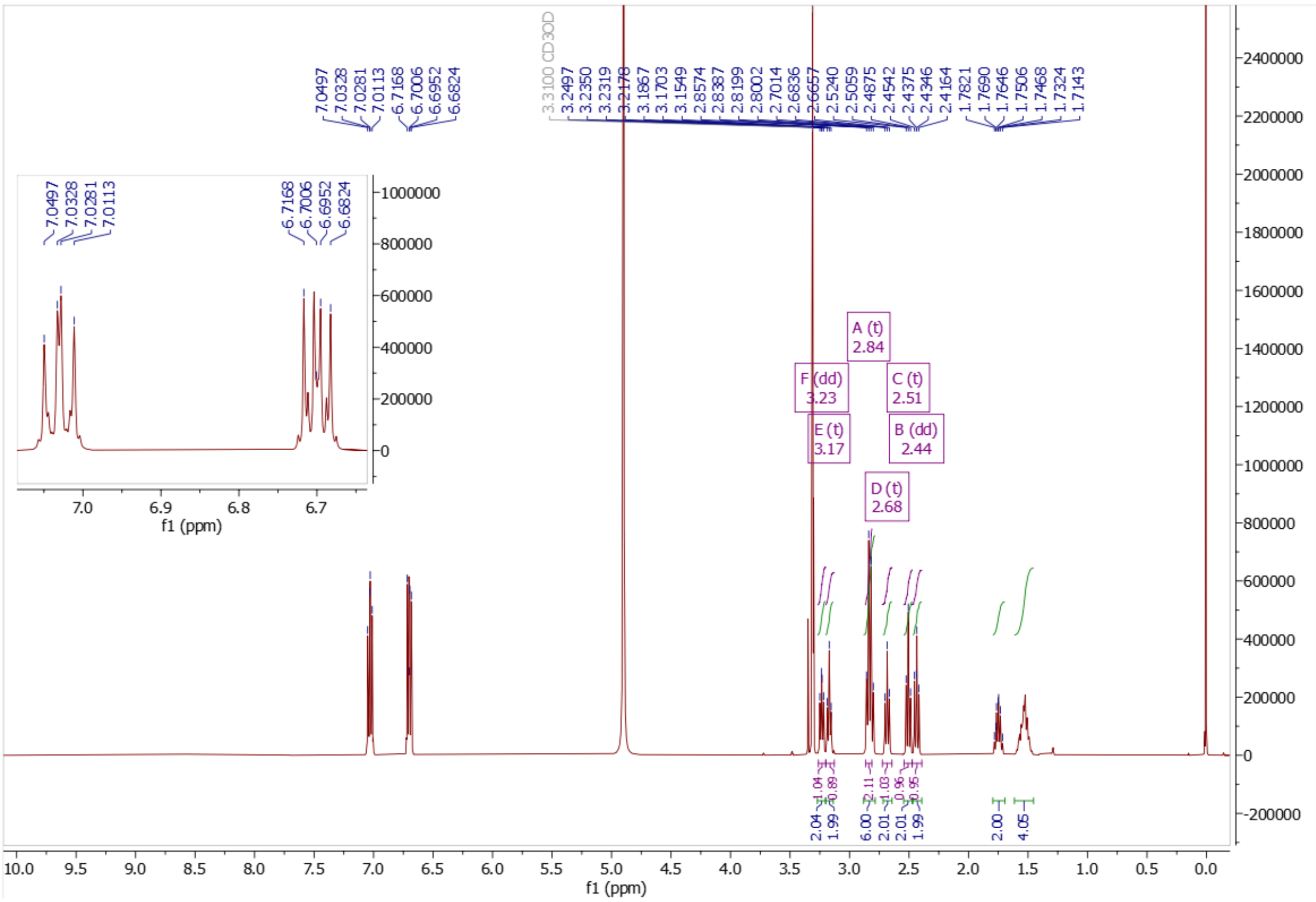

Figure S7. ^{1}H NMR (400 MHz, CD_3OD) spectrum of synthesized *N*[1],*N*[10]-bis(dihydro-*p*-coumaroyl)-spermidine (metabolite **3**).

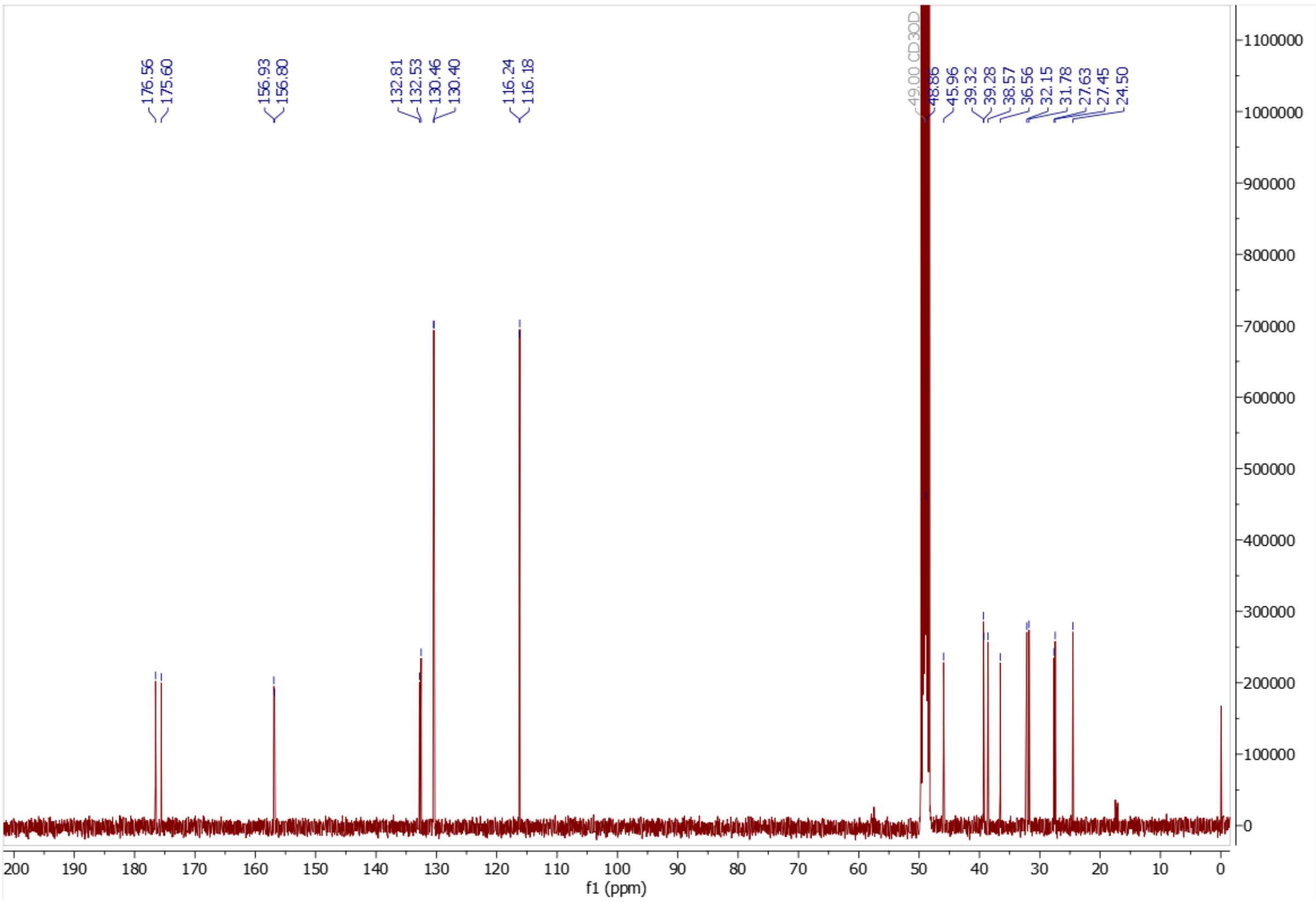

Figure S8. ^{13}C NMR (100 MHz, CD_3OD) spectrum of synthesized *N*[1],*N*[10]-bis(dihydro-*p*-coumaroyl)-spermidine (metabolite **3**).

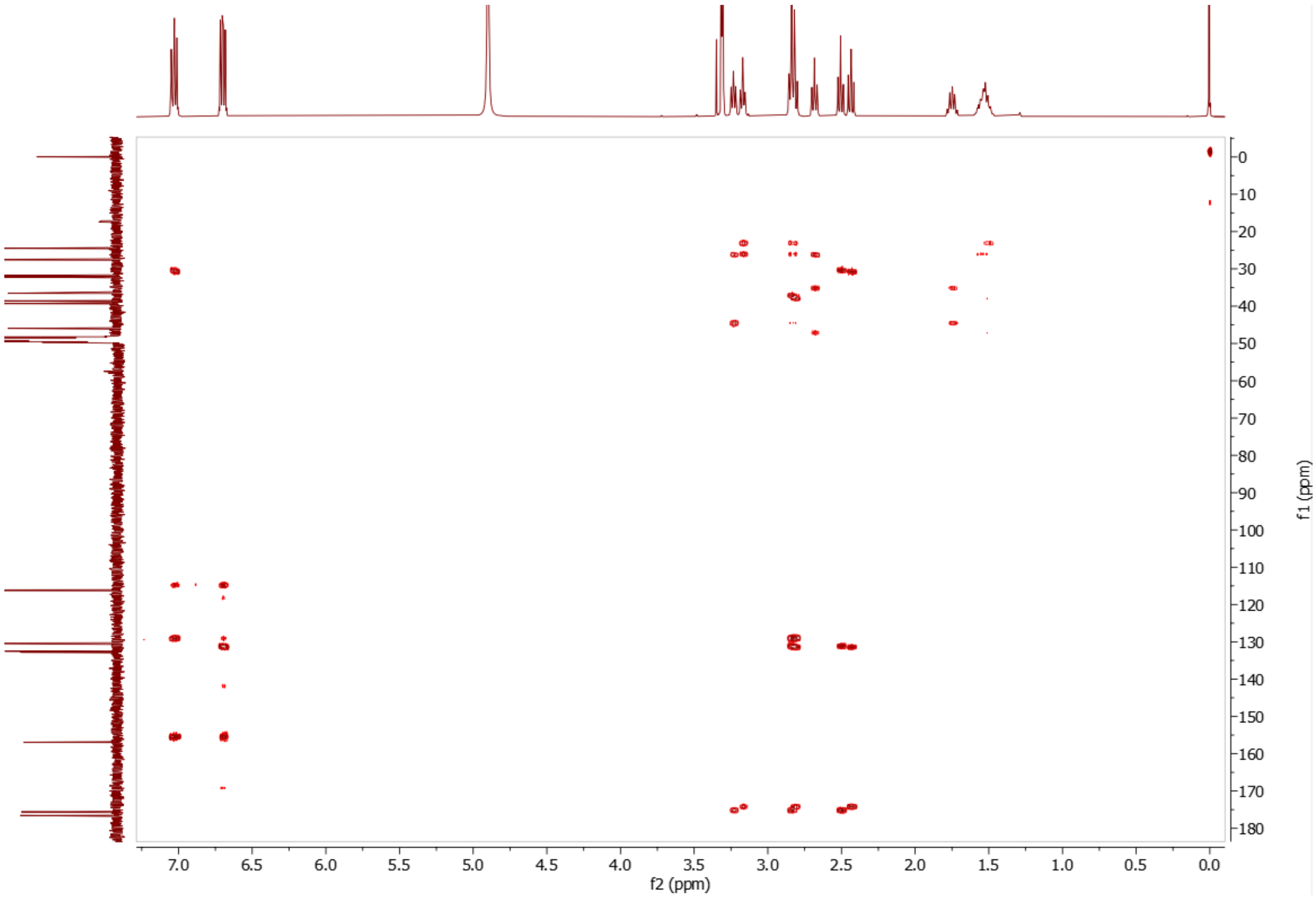

Figure S9. HMBC spectrum of synthesized *N*[1],*N*[10]-bis(dihydro-*p*-coumaroyl)-spermidine (metabolite **3**).
